# PROTOCOL: Abortion and mental health outcomes: A systematic review and meta‐analysis

**DOI:** 10.1002/cl2.1410

**Published:** 2024-05-21

**Authors:** Julia H. Littell, Sarah Young, Therese D. Pigott, M. Antonia Biggs, Trine Munk‐Olsen, Julia R. Steinberg

**Affiliations:** ^1^ Graduate School of Social Work and Social Research Bryn Mawr College Bryn Mawr Pennsylvania USA; ^2^ Hunt Library Carnegie Mellon University Pittsburgh Pennsylvania USA; ^3^ College of Education and Human Development Georgia State University Atlanta Georgia USA; ^4^ Advancing New Standards in Reproductive Health, Department of Obstetrics, Gynecology & Reproductive Sciences, School of Medicine University of California San Francisco Oakland California USA; ^5^ Department of Psychiatry University of Southern Denmark Odense Denmark; ^6^ Department of Family Science, School of Public Health University of Maryland College Park Maryland USA

**Keywords:** abortion, mental health, meta‐analysis, systematic review

## Abstract

This is a protocol for a systematic review and meta‐analysis of research on mental health outcomes of abortion. Does abortion increase the risk of adverse mental health outcomes? That is the central question for this review. Our review aims to inform policy and practice by locating, critically appraising, and synthesizing empirical evidence on associations between abortion and subsequent mental health outcomes. Given the controversies surrounding this topic and the complex social, political, legal, and ideological contexts in which research and reviews on abortion are conducted, it is especially important to conduct this systematic review and meta‐analysis with comprehensive, rigorous, unbiased, and transparent methods. We will include a variety of study designs to enhance understanding of studies' methodological strengths and weaknesses and to identify potential explanations for conflicting results. We will follow open science principles, providing access to our methods, measures, and results, and making data available for re‐analysis.

## 1 BACKGROUND

### 1.1 The problem, condition, or issue

Abortion, the voluntary termination of a pregnancy, has long been a subject of controversy. Ideological and legal debates on this issue reflect different views about when human life begins and the rights and autonomy of women versus the rights of an embryo or fetus. In recent decades, a newer controversy has centered on whether abortion has adverse effects on women's mental health (Lee, 2003; Major et al., 2009; Siegel, 2008). In 1989, a review by the U.S. Surgeon General concluded that the quality of available evidence was weak and “the data do not support the premise that abortion does or does not cause or contribute to psychological problems” (Koop, 1989, p. 174). Since then, many studies on this topic have been completed; these studies use divergent methodologies and reach different conclusions. Previous reviews of this body of research have not settled the controversy.

Claims that abortion is harmful to women's mental health have influenced policies around the globe, including abortion bans, gestational limits, mandated waiting periods, and counseling laws. Although these claims are contested, they have influenced recent landmark court decisions and legislation to restrict access to abortion, particularly in the USA. These claims appeared in arguments before the US Supreme Court in the 2022 case (*Dobbs v Jackson Womenʼs Health Organization*) that eliminated the constitutional right to abortion in the USA, and in a 2023 US District Court ruling (Kacsmaryk, 2023) that sought to overturn the US Food and Drug Administration's 2000 approval of mifepristone, one of the most widely used abortion medications. In the USA, some states require health care providers to inform patients that abortion has adverse mental health consequences (National Academies of Sciences, Engineering, and Medicine [NASEM], 2018).

Does abortion increase the risk of adverse mental health outcomes? That is the central question for this systematic review and meta‐analysis. Our review aims to inform policy and practice, by locating, critically appraising, and synthesizing empirical evidence on associations between abortion and subsequent mental health outcomes. Given the controversies surrounding this topic and the complex social, political, legal, and ideological contexts in which research and reviews on abortion are conducted, it is especially important to conduct this systematic review and meta‐analysis with rigorous, unbiased, and transparent methods. We will include a variety of study designs to enhance understanding of studies' methodological strengths and weaknesses and to identify potential explanations for conflicting results.

Throughout this protocol, we use the terms “women” and “persons” somewhat interchangeably, to refer to females, cisgender women, transgender men, and gender non‐binary individuals who are capable of pregnancy and childbirth.

Because the vast majority of abortions result from unwanted or mistimed pregnancies (Kost et al., 2023), we begin with a brief discussion of pregnancy intentions and abortion. Then we describe legal restrictions on abortion.

#### 1.1.1 Pregnancy intentions and abortion

Unintended pregnancies are usually defined to include unplanned, mistimed, and unwanted pregnancies. The best available data suggest that almost half (48%) of all pregnancies worldwide are unintended and 61% of unintended pregnancies end in abortion, although rates vary considerably by region and demographic characteristics (Bearak et al., 2020, 2022). Unintended pregnancy and abortion rates tend to be higher in low‐ and middle‐income countries and among people living in poverty in high income countries (Bearak et al., 2020; Finer & Zolna, 2016).

Unintended pregnancy is associated with an array of negative health, economic, social, and psychological outcomes for women and children (National Collaborating Centre for Mental Health [NCCMH], 2011; Nelson et al., 2022). Until recently, the incidence of unintended pregnancy has been treated as one of the “most essential health status indicators in the field of reproductive health” (Finer & Zolna, 2011, p. 478).

Measures of unintended pregnancy are usually binary and retrospective; they do not capture the complexity of pregnancy desires (including uncertainty and ambivalence) and they conflate pregnancy planning with wanted pregnancies (i.e., unplanned and mistimed pregnancies may or may not be wanted; Auerbach et al., 2023, Kost et al., 2023). For purposes of research on abortion, information on unwanted and mistimed pregnancies is more relevant than data on pregnancy planning or composite measures of pregnancy intentions. Nevertheless, many studies use imperfect measures of pregnancy intentions.

#### 1.1.2 Legal restrictions on abortion

Access to safe and legal abortion varies widely by policy context and whether abortion is banned, highly restricted, or stigmatized (Ganatra et al., 2017). Legal restrictions on abortion vary across jurisdictions and are often based on gestational duration and the reason for abortion. In some jurisdictions, abortions are only allowed to preserve the pregnant person's life or health (sometimes including mental health), for socioeconomic reasons, or in cases of rape, incest, or fetal anomaly (Remez et al., 2020). Some jurisdictions require parental consent for minors, require abortion procedures to be performed by a licensed physician, require involvement of a second physician, require abortions to be performed in a hospital after a certain gestational age, require waiting periods, and so forth. In some places, providers are mandated to provide counseling before an abortion, and sometimes this includes warning patients of physical and mental health consequences of abortion (National Academies of Sciences, Engineering, & and Medicine [NASEM], 2018).

The global trend toward reducing legal restrictions and expanding legal access to abortion continued over the past decade (Remez et al., 2020), but legal restrictions on abortion have increased in the USA for at least the past 10 years (Guttmacher Institute, 2021). Laws that restrict abortion reduce but do not eliminate its practice (Remez et al., 2020).

### 1.2 The intervention: Abortion

When abortion is the result of an intervention, it is sometimes called “induced abortion.” In contrast, miscarriage or pregnancy loss (sometimes referred to as “spontaneous abortion”) occurs without any intervention in approximately 15% of all recognized pregnancies (Quenby et al., 2021). We use the term “abortion” to refer to any voluntary termination of pregnancy and not to miscarriage or pregnancy loss.

Abortion is safe and effective when carried out by methods recommended by the World Health Organization (Lohr et al., 2014; National Academies of Sciences, Engineering, and Medicine [NASEM], 2018; World Health Organization, 2022, 2023), including use of medications approved for this purpose (mifepristone and/or misoprostol, and methotrexate) and/or approved medical procedures such as uterine aspiration, dilation and curettage, dilation and evacuation, or dilation and extraction). The former are termed medication abortions and the later are called procedural abortions (Upadhyay et al., 2023). Both types of abortion are included in this review.

Self‐managed abortions can be very safe and effective with the use of misoprostol or mifepristone medications (Moseson et al., 2020, 2022, 2023), but the same cannot be said for self‐managed abortions that use physical force or other (potentially harmful) substances (Ralph et al., 2020; Remez et al., 2020). Therefore, this review will include studies of abortions that are self‐managed with mifepristone and/or misoprostol, but it will not include studies of other self‐managed or non‐medical abortion procedures.

Safe and effective abortion methods depend, in part, on gestational duration. Thus, womens' experiences of abortion are likely to be affected by abortion method and gestational duration, along with characteristics of the socioeconomic, cultural, legal, health care, and relational contexts in which abortions occur (Russo, 2014). In high‐income countries at least half of all abortions are medication abortions, at least 90% of all abortion occur within the first 13 weeks of pregnancy, and two‐thirds occur within 9 weeks (Popinchalk & Sedgh, 2019). Global data on gestational duration at abortion are scarce.

Pregnant persons who do not have medication or procedural abortions may experience live births (full‐term or premature deliveries) or fetal loss (miscarriages or stillbirths), or may end the pregnancy on their own without the use of approved medications or the support of a health professional.

### 1.3 How and why abortion might relate to mental health outcomes

**Figure 1:**
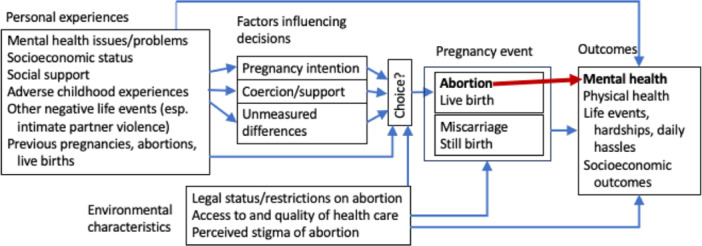
Abortion and mental health outcomes: two theories of change. Theory 1 (illustrated with a red arrow): Abortion is a traumatic event that increases risks of negative mental health outcomes. Theory 2 (illustrated with blue arrows): Abortion is confounded with other factors that affect mental health. Pregnancy events (including but not limited to abortion) may moderate or mediate influences of personal experiences and environmental characteristics on outcomes. Abortion does not have consistent, independent (direct) effects on mental health outcomes.

As illustrated in Figure 1, there are at least two competing explanations of how and why abortion might be related to subsequent mental health outcomes. We refer to the first as abortion‐trauma theory and the second as a co‐occurring risks perspective.

#### 1.3.1 Abortion‐trauma theory and “post‐abortion syndrome”

One theory is that “abortion is a uniquely traumatic experience because it involves a human death experience, specifically, the intentional destruction of one's unborn child and the witnessing of a violent death, as well as a violation of parental instinct and responsibility, the severing of maternal attachments to the unborn child, and unacknowledged grief” (Major et al., 2009, p. 866). Rue and Speckhard (Rue & Speckhard, 1992; Rue, 2014; Speckhard & Rue, 1992; Speckhard, 1985) suggested that the traumatic experience of abortion could lead to serious mental health problems, which they termed “post‐abortion syndrome” (PAS). PAS was conceptualized as a specific form of posttraumatic stress disorder (PTSD) “comparable to the symptoms experienced by Vietnam veterans, including symptoms of trauma, such as flashbacks and denial, and symptoms such as depression, grief, anger, shame, survivor guilt, and substance abuse” (Major et al., 2009, p. 866). PAS is not recognized as a mental disorder in the DSM5 or ICD‐10.

The abortion‐trauma theory was originally developed from Speckhard's (1985) interviews with 30 women who had “highly stressful” abortions in the past 1 to 25 years, including some who had illegal abortions, 14 women who had second trimester abortions, and 1 who had an abortion in the third trimester (Major et al., 2009). As indicated above, some subsequent studies seemed to support this view. For example:

- lifetime measures of abortion were correlated with lifetime measures of mental health problems (Coleman et al., 2009; Steinberg & Finer, 2011);
- lifetime history of abortion or miscarriage were correlated with lifetime measures of substance use disorders and affective disorders in 21 year‐old women (Dingle et al., 2008);
- after adjustment for some confounding variables (but not whether pregnancy was wanted or mistimed), abortion was associated with an increase in the risk of mental disorders (Fergusson et al., 2006, 2008); and
- one meta‐analysis estimated that “women who had undergone an abortion experienced an 81% increased risk of mental health problems, and nearly 10% of the incidence of mental health problems was shown to be directly attributable to abortion” (Coleman, 2011, p. 183).

Abortion‐trauma theory has been vigorously disputed by Stotland, 1992 and others (Charles et al., 2008; Major et al., 2009; National Academies of Sciences, Engineering, &and Medicine [NASEM], 2018; National Collaborating Centre for Mental Health [NCCMH], 2011; Steinberg & Russo, 2009) who noted that the evidence used to support a causal link between abortion and subsequent mental health disorders or symptoms is marred by serious methodological problems including use of highly selected samples, inadequate comparison groups, inadequate conceptualization and control of relevant variables, unreliable outcome measures, inappropriate statistical analyses, and errors of interpretation including misattribution of causal effects (Robinson et al., 2009; Steinberg & Finer, 2011).

Some evidence seems to contradict the abortion‐trauma view. For example, a prospective, multi‐site cohort study in Sweden found that few women developed PTSD or Posttraumatic Stress Symptom (PTSS) after abortion, and most cases of PTSD or PTSS were due to trauma experiences unrelated to induced abortion (Wallin Lundell et al., 2013). A recent, prospective longitudinal cohort study of patients who sought abortion at 30 abortion clinics in the USA (The Turnaway Study) showed that those who received abortions were at no higher risk of PTSD than patients who were denied abortions (Biggs et al., 2016); further, there were no long‐term differences in perceived stress, although patients who were denied abortions experienced more perceived stress immediately afterward than did patients who had abortions (Harris et al., 2014).

#### 1.3.2 Co‐occurring risks: A psychological and sociological perspective

A second explanation for how and why abortion might relate to mental health outcomes is that abortion is confounded with other factors that impact mental health, but abortion per se does not have an independent effect on mental health outcomes. This view is derived from three complementary conceptual frameworks, which Major et al. (2009) identified as the stress and coping, sociocultural, and co‐occurring risk perspectives (we include all three within a broader co‐occurring risks perspective).

The stress‐and‐coping perspective suggests that abortion is one of many stressful life events, such as childbirth, but abortion co‐occurs with other potentially stressful events, such as unintended or unwanted pregnancy. Adaptations to such compounded, stressful events are highly varied and are likely influenced by the person's cognitive appraisal of their situation, their perceived ability to cope with it, and available social support (Major et al., 2009).

The sociocultural perspective suggests that the perceived stigma of experiences like abortion can lead to negative psychological outcomes. Perceived stigma and “internalized stigma” (shame and guilt) have been linked to a variety of cognitive, emotional, and behavioral problems (see Major et al., 2009). Further, societal messages about what women should do regarding unintended or unwanted pregnancies (the extent to which abortion is stigmatized or normalized) or should experience regarding abortion (e.g., relief or shame and guilt) can become self‐fulfilling expectations. Thus, social stigma, internalized stigma, and sociocultural norms can have important mediating roles in the relationship between abortion and mental health outcomes.

Finally, a number of potent risk factors for mental health problems also increase the likelihood of unwanted pregnancy, and thus the likelihood of desiring an abortion. As mentioned above, unintended pregnancy is associated with an array of negative health, economic, social, and psychological outcomes for women and children (National Collaborating Centre for Mental Health [NCCMH], 2011; Nelson et al., 2022). Thus, “systemic, social, and personal factors that are precursors to unintended pregnancy … place women at risk for having abortions and/or predispose them to experience mental health problems regardless of pregnancy and its resolution” (Major et al., 2009, p. 868). Epidemiological studies show that women who have abortions are disproportionately at risk of interpersonal violence and other types of violence (National Academies of Sciences, Engineering, and Medicine [NASEM], 2018).

Other confounding variables include socioeconomic status, race and ethnicity, smoking, and substance use (National Academies of Sciences, Engineering, and Medicine [NASEM], 2018). “Thus, the myriad of disadvantages, such as low socioeconomic status, lack of education, and violence, putting women at risk for unintended pregnancy are also associated with having an abortion” (Steinberg & Russo, 2009, p. 500). It may be the co‐occurring risk factors, not abortion per se, that account for subsequent mental health outcomes. Indeed, one review found that:

- rates of mental health problems for women with an unwanted pregnancy were the same regardless of whether they had an abortion or gave birth,
- the most reliable predictor of post‐abortion mental health problems was having a history of mental health problems before the abortion, and
- factors associated with increased rates of mental health problems for women in the general population following birth and following abortion were similar (National Collaborating Centre for Mental Health [NCCMH], 2011).

As depicted in Figure 1, pregnancy, pregnancy‐related events, and other stressful life events can have diverse direct and/or indirect effects on mental health outcomes (e.g., postpartum depression) as well as outcomes related to patients' physical health, daily hardships, stressors, and socioeconomic variables. When background and contextual factors are not adequately taken into account, abortion may act as a proxy for exposure to other risk factors (e.g., socioeconomic disadvantage, prior mental health issues, social stigma, exposure to violence including sexual assault), leading to spurious associations between abortion and mental health outcomes.

Russo noted that, although the predictors of problems after abortion or childbirth are similar, “it is reasonable to assume that there is something different about women who have unwanted pregnancies and opt for abortion over delivery” (Russo, 2014, p. 285). Unmeasured differences between these groups may be important.

Pregnancy and related events may mediate or moderate effects of personal background characteristics and environmental conditions on mental health outcomes. For example, pregnancy and childbirth or abortion might exacerbate or ease ongoing mental health issues.

It is reasonable to assume that there are complex interactions and reciprocal effects among predictors and outcome variables in this conceptual model, so that mental health outcomes affect and are affected by other outcomes. Further, the direction of influence between abortion and mental health is not always clear. Some studies show that mental health problems tend to peak immediately before an abortion, not afterward (Major et al., 2000). One study found that, compared with those who give birth, patients who had an abortion reported higher rates of mood disorders before the procedure (Steinberg et al., 2014). “Therapeutic abortion” is sometimes provided to preserve patients' mental health in cases with “psychological distress or mental suffering due to unwanted pregnancy and responsibility for childcare, or … anticipated serious fetal impairment” (Cook et al., 2006, p. 185). Thus, abortion can be viewed as both a predictor and an outcome of mental health problems.

In the overarching co‐occurring risks model (which incorporates the stress and coping and sociocultural perspectives) shown in Figure 1, (1) confounding variables play an important role in understanding the relationship between abortion and mental health outcomes, and (2) pregnancy events may moderate or mediate effects of personal and environmental characteristics on outcomes, but (3) abortion itself does not have consistent or independent (direct) effects on mental health outcomes. This theory predicts that having an abortion does not increase a patient's risk of problems such as depression, anxiety, and/or PTSD (National Academies of Sciences, Engineering, and Medicine [NASEM], 2018). Criticisms of this approach might focus on its complexity.

### 1.4 Why it is important to do this review

As described above, there are conflicting views about the relationship between abortion and subsequent mental health outcomes. Conflicting information has been provided to judicial and legislative bodies that are responsible for legal regulations on abortion, as well as to patients in some nations and states. A rigorous systematic review and meta‐analysis is needed to provide a credible, up‐to‐date synthesis of research on this topic.

#### 1.4.1 Primary studies of abortion and mental health outcome

Many studies have examined associations between abortion and risks of post‐traumatic stress and other anxiety disorders, depression and other mood disorders, suicidal ideation and suicidal behavior, substance use disorders, and/or a composite measure of any mental disorder (e.g., Biggs et al., 2015, 2016, 2017; Coleman et al., 2009; Crandell, 2012; Fergusson et al., 2006, 2008; Foster, 2020; Gissler et al., 1996; Munk‐Olsen et al., 2011; Pedersen, 2007, 2008; Van Ditzhuijzen et al., 2017; Wallin Lundell et al., 2013). A few studies have looked at abortion in relation to eating disorders (Linna et al., 2013), sleep disorders (Reardon & Coleman, 2006), sexual dysfunction (Coleman et al., 2008), and psychotic disorders (Brewer, 1977; David et al., 1981; Gilchrist et al., 1995). Abortion has also been studied in relation to rates of outpatient mental health treatment (Coleman et al., 2002; Studnicki et al., 2023), psychiatric hospitalization (Reardon et al., 2003; Studnicki et al., 2023), and reported use of psychotropic medication for mental disorders (Steinberg et al., 2018).

Studies with parallel cohorts have included different types of comparison groups (sometimes called reference groups or control groups), including: people who had miscarriages (Broen et al., 2005; Dingle et al., 2008), people who sought but did not receive abortions (Biggs et al., 2017; Foster, 2020); people who had unintended pregnancies but carried them to term (Gilchrist et al., 1995); people who had live births (David et al., 1981; Studnicki et al., 2023), and birth cohorts from a general population including people who were never pregnant (Munk‐Olsen et al., 2011; Pedersen, 2007, 2008; Van Ditzhuijzen et al., 2017).

#### 1.4.2 Previous reviews of research on this topic

Our preliminary search identified several nonsystematic, narrative reviews of research on abortion and women's mental health (American Psychological Association [APA] Task Force on Mental Health and Abortion, 2008; Coleman et al., 2005; Russo, 2014; Major et al., 2009; National Academies of Sciences, Engineering, and Medicine [NASEM], 2018; Robinson et al., 2009; Steinberg et al., 2009; Steinberg, 2011; Grupo Médico por el Derecho a Decidir – Columbia, 2011), as well as five systematic reviews and/or meta‐analyses on this topic (Bellieni & Buonocore, 2013; Charles et al., 2008; Coleman, 2011; Fergusson et al., 2013; National Collaborating Centre for Mental Health [NCCMH], 2011).

In Table 1, we assess the adequacy of previous systematic reviews and meta‐analyses, using the AMSTAR tool (for Assessment of Multiple SysTemAtic Reviews; Shea et al., 2007). We use the version of AMSTAR that was already available when these reviews were published (a latter version, AMSTAR 2, includes additional criteria that none of these reviews addressed; Shea et al., 2017). Our analysis of these reviews is based solely upon public *a priori* protocols (if available) and published reports.

**Table 1:** Assessment of previous systematic reviews and meta‐analyses (adapted from AMSTAR; Shea et al., 2007).

	Charles et al. (2008)	Coleman (2011)	NCCMH (2011)	Fergusson et al. (2013)	Bellieni and Buonocore (2013)
Number of studies included	21 studies with comparison groups, 5 without comparison groups	22 studies (publications) based on 14 distinct databases	15 studies in section on comparative risks	4 studies (14 analyses, 8 publications; reanalysis of data from Coleman (2011) and NCCMH (2011)	30 studies with comparison groups
**1. Was an *a priori* design provided?** The research question and inclusion criteria should be established before the conduct of the review.	**No**	**No**	**No**	**No**	**No**
**2. Was there duplicate study selection and data extraction?** There should be at least two independent data extractors and a consensus procedure for disagreements should be in place.	**Yes**	**No**	**Yes**	**No**	**No**
**3. Was a comprehensive literature search performed?** At least two electronic sources should be searched. The report must include years and databases used (e.g., Central, EMBASE, and MEDLINE). Keywords and/or MESH terms must be stated and where feasible the search strategy should be provided. All searches should be supplemented by consulting current contents, reviews, textbooks, specialized registers, or experts in the particular field of study, and by reviewing references in studies found.	**No**	**No**	**Yes**	**No**	**No**
**4. Was the status of publication (i.e., grey literature) avoided as an inclusion criterion?** The authors should state that they searched for reports regardless of their publication type. The authors should state whether or not they excluded any reports (from the systematic review), based on their publication status, language, and so forth.	**No** (limited to published studies)	**No** (limited to studies published in English in peer‐reviewed journals, 1995–2009)	**Yes** (searched for unpublished studies via contacts with experts; limited to English reports, 1990–2011)	**No**	**No** (limited to studies published 1995–2011)
**5. Was a list of studies (included and excluded) provided?** A list of included and excluded studies should be provided.	**No** (excluded studies not listed)	**No**	**Yes**	**No**	**Yes**
**6. Were the characteristics of the included studies provided?** In an aggregated form such as a table, data from the original studies should be provided on the participants, interventions, and outcomes. The ranges of characteristics in all the studies analyzed, for example, age, race, sex, relevant socioeconomic data should be reported.	**Yes**	**No**	**Yes**	**Yes**	**Yes**
**7. Was the scientific quality of the included studies assessed and documented?** *A priori* methods of assessment should be provided.	**Unclear** (overall quality ratings were not documented)^a^	**No**	**Yes** (use of 3 NICE methodology checklists, overall quality ratings,^a^ and GRADE)	**Unclear** (used overall study quality ratings from NCCMH review)^a^	**Unclear** (use of an overall design rating)
**8. Was the scientific quality of the included studies used appropriately in formulating conclusions?** The results of the methodological rigor and scientific quality should be considered in the analysis and the conclusions of the review, and explicitly stated in formulating recommendations.	**No** (use of overall quality ratings and vote counting)	**No**	**Yes** (used GRADE)	**Yes**	**No**
**9. Were the methods used to combine the findings of studies appropriate?** For the pooled results, a test should be done to assess their homogeneity (i.e., Chi‐squared test for homogeneity, *I* ^2^). If heterogeneity exists a random effects model should be used and/or the clinical appropriateness of combining should be taken into consideration (i.e., is it sensible to combine?).	**No** (used vote counting)	**No** (meta‐analysis included dependent effect sizes from overlapping samples)	**Unclear** (heterogeneity tests were used to determine whether meta‐analysis was appropriate)	**Unclear** (used vote‐counting in addition to meta‐analysis)	**No** (used vote‐counting)
**10. Was the likelihood of publication bias assessed?** Assessment of publication bias should include a combination of graphical aids (e.g., funnel plot) and/or statistical tests (e.g., Egger regression test).	**No**	**No**	**No**	**No**	**No**
**11. Was the conflict of interest (COI) stated?** Potential sources of support should be clearly acknowledged in both the systematic review and the included studies.	**Partially** (support for the review was acknowledged, but there was no COI statement and COI in included studies was not reported)	**No**	**Partially** (interests of reviewers were documented, but COI in included studies was not)	**Partially** (support for the review was acknowledged, authors declared they had no conflicts, but they did not document COI in included studies)	**No** (authors reported no COI, but sources of support were not reported for the review or for included studies)
**Reviewers' conclusions**	“A clear trend emerges from this systematic review: the highest quality studies had findings that were mostly neutral, suggesting few, if any, differences between women who had abortions and their respective comparison groups in terms of mental health sequelae. Conversely, studies with the most flawed methodology found negative mental health sequelae of abortion” (p. 436).	“Women who had undergone an abortion experienced an 81% increased risk of mental health problems, and nearly 10% of the incidence of mental health problems was shown to be directly attributable to abortion” (p. 183).	“The rates of mental health problems for women with an unwanted pregnancy were the same whether they had an abortion or gave birth. An unwanted pregnancy was associated with an increased risk of mental health problems. The most reliable predictor of post‐abortion mental health problems was having a history of mental health problems before the abortion. The factors associated with increased rates of mental health problems for women in the general population following birth and following abortion were similar. There were some additional factors associated with an increased risk of mental health problems specifically related to abortion, such as pressure from a partner to have an abortion and negative attitudes towards abortions in general and towards a woman's personal experience of the abortion” (p. 8).	“There is no available evidence to suggest that abortion has therapeutic effects in reducing the mental health risks of unwanted or unintended pregnancy. There is suggestive evidence that abortion may be associated with small to moderate increases in risks of some mental health problems” (p. 819).	“Fetal loss seems to expose women to a higher risk for mental disorders than childbirth; some studies show that abortion can be considered a more relevant risk factor than miscarriage…” (p. 301).

^a^ *A priori* methods for study quality assessment were not used. Overall quality ratings included categories that were not clearly defined (e.g., “appropriate” comparison groups) and “other methodological factors” that were “not … uniformly considered across studies” (Charles et al., 2008, p. 437).

None of these reviews followed (or cited) any of the published guidelines for the conduct and reporting of systematic reviews and meta‐analyses that were available at the time (e.g., Institute of Medicine, 2011; Moher et al., 1999, 2009; Stroup, 2000), nor did they reflect (or cite) the substantial body of theoretical, statistical, and methodological work upon which these guidelines were based (see Cooper & Hedges, 1994; Cooper et al., 2009; Hedges & Olkin, 1985; Higgins & Green, 2011; Light & Pillemer, 1984; Lipsey & Wilson, 2001; Littell et al., 2008; Petticrew & Roberts, 2006; Rothstein et al., 2005). Instead, most reviews cited prior reviews, a common practice in many fields of research, which leads to repetition of old practices, not necessarily best practices. Thus, these reviews did not reflect major developments in research synthesis methods through the early 2000s (or later), including efforts to discourage the use of publication status as a criterion for inclusion in a review (in light of evidence of systematic reporting, publication, and dissemination biases; Rothstein et al., 2005; Song et al., 2000, 2009, 2010; Stroup, 2000) and concerns about the use of overall study quality scores or ratings, which conflate potentially unrelated sources and types of biases (Jüni, 1999, 2001; Stroup, 2000; Wells & Littell, 2009). Indeed, several reviews systematically excluded unpublished studies and used overall study quality ratings (see Table 1).

As shown in Table 1, none of these reviews provided a public protocol in advance of conduct of the review to improve transparency and reduce bias (see Stewart et al., 2012). None of these reviews assessed the likelihood of publication bias or its potential impact on results (see Rothstein et al., 2005).

Only one review (National Collaborating Centre for Mental Health [NCCMH], 2011) conducted a comprehensive literature search (one that could retrieve relevant unpublished studies) and avoided systematic exclusion of unpublished studies. The other reviews shown in Table 1 used convenience samples of published studies, which are likely to produce biased results (Song et al., 2000, 2009, 2010).

The review conducted by the UK National Collaborating Centre for Mental Health (National Collaborating Centre for Mental Health [NCCMH], 2011) at the Royal College of Psychiatrists (RCP), underutilized meta‐analysis. It relied on heterogeneity tests to determine whether meta‐analysis was “appropriate,” instead of using random effects models for heterogeneous sets of studies (see Borenstein et al., 2009, 2010). As a result, this review overutilized narrative synthesis, which is demonstrably less accurate than meta‐analysis (Bushman & Wells, 2001; Cooper & Rosenthal, 1980).

Only two reviews (Charles et al., 2008; National Collaborating Centre for Mental Health [NCCMH], 2011) conducted duplicate study selection and data extraction, which can reduce bias and error (Buscemi et al., 2006; Gøtzsche et al., 2007).

Several reviews used overall study quality ratings that included undefined criteria (e.g., “appropriate” comparison groups) and “other methodological factors” that were “not … uniformly considered across studies” (Charles et al., 2008, p. 437). This approach was adopted in the National Collaborating Centre for Mental Health (NCCMH) (2011) review (alongside other critical appraisal methods) and overall quality ratings from the National Collaborating Centre for Mental Health (NCCMH) (2011) review were used in the Fergusson et al. (2013) review. As indicated above, overall quality scores conflate distinct sources and types of bias, which may have different effects on results; thus, these scores lack construct and discriminant validity.

As shown in Table 1, several reviews used vote‐counting methods, which tally the number of studies according to the direction and statistical significance of their results. This is not a valid synthesis method, because such tallies are imprecise (uninformed by the magnitude of the effect size, sample size, or heterogeneity) and potentially misleading (Bushman, 1994; Grainger et al., 2022; Hedges & Olkin, 1985). Charles et al. (2008) used a vote‐counting procedure to tally studies according to (a) the direction and significance of their findings and (b) overall quality ratings, thereby combining an invalid synthesis method (vote‐counting) with unreliable and invalid ratings.

The Coleman (2011) meta‐analysis (cited above) did not meet any of the AMSTAR criteria (Littell & Coyne, 2012). Further, it included multiple dependent effect sizes derived from the same data sets, and synthesized these effects as if they were independent estimates. This approach produces results that are not reliable, accurate, or meaningful.

Fergusson et al. (2013) reanalyzed data from the Coleman (2011) and National Collaborating Centre for Mental Health (NCCMH) (2011) reviews, limiting meta‐analysis to effect sizes from non‐overlapping samples (to manage dependent effect sizes properly).

Bellieni and Buonocore (2013) provided separate (vote‐counting) analyses which compared abortion with different alternatives: childbirth, unplanned childbirth, or miscarriage.

Table 1 shows that these five reviews drew substantially different conclusions from research on associations between abortion and subsequent mental health outcomes. Some reviews concluded that abortion increased the risks of subsequent mental health problems, while others found that these problems were associated with unintended pregnancy and prior mental health problems, not abortion per se, and a third set of conclusions suggests that results vary based on study quality. In any case, serious methodological flaws in each of these reviews undermines confidence in their conclusions.

There are no reviews on this topic that meet current published guidelines for the conduct of systematic reviews and meta‐analysis (e.g., Page et al., 2021). A rigorous systematic review and meta‐analysis is needed to provide robust and reliable critical appraisal, analyses, and syntheses of the empirical evidence on the mental health outcomes of abortion. Our review aims to meet that need.

#### 1.4.3 Relevance for policy and practice

Better understanding of associations between abortion and mental health outcomes could inform policy and practice regarding screening and treatment for mental health problems in relation to reproductive health care.

### 1.5 Our approach: Guiding principles and assumptions

To produce credible evidence on this controversial topic, our review must be as rigorous and transparent as possible. To ensure rigor, we will follow the Campbell Collaboration's standards for the conduct and reporting of systematic reviews and meta‐analyses (The Methods Coordinating Group of the Campbell Collaboration, 2019a, 2019b), rely on empirical evidence on the best ways to minimize bias and error in systematic reviews and meta‐analysis, and utilize the best available review methodologies. To ensure transparency, we will clearly define key constructs and measures. We will carefully document our procedures, including any changes in plans. We will follow open science principles, providing access to our methods, measures, and results on the website: https://osf.io/g6bju/. To minimize bias and error, we have developed and will follow steps (described in Section 3 below) that have been shown to reduce bias and error in research reviews; these plans include steps to report and manage any potential conflicts of interest.

**Figure 2:**
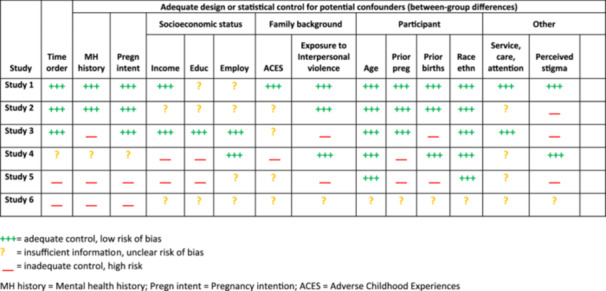
Sample confounder matrix. ACES, Adverse Childhood Experiences; MH history, Mental health history; Pregn intent, Pregnancy intention. *Source*: adapted from Petersen et al., 2022.

In constructing this review, we have made use of the logic of causal inference and understanding of various counterfactual models (Shadish et al., 2002). For reasons described above, it is difficult to assess the effects of abortion on mental health outcomes. Randomized controlled studies on this topic are not ethical. However, we can synthesize data from observational studies that include the prerequisites for causal inference: correlation, time order, and ruling out other plausible explanations (Shadish et al., 2002). To assess correlations, abortion must be studied as a variable, not a constant (i.e., compared with a non‐abortion group). To establish time order, abortion must precede the measurement of mental health outcomes. One of our challenges will be assessing the extent to which studies are able to establish time order. Another challenge will be assessing the extent to which studies are able to rule out other plausible explanations, including unmeasured pre‐existing differences between groups of people who had abortions and those who did not (see Figures 1 and 2).

In this protocol, we refer to variables that may be associated with both abortion and mental health outcomes as potential *confounding* variables (or confounds), especially if there are uncontrolled *within‐study* differences on these variables between abortion and non‐abortion groups. Between‐study differences (e.g., use of different types of comparison groups, use of statistical controls for prior measures of mental health) are treated as potential *moderators* of the association between abortion and mental health outcomes. We will use meta‐analysis to synthesize study‐level (aggregated) data on confounds and moderators; we will not use meta‐analytic structural equation models to investigate whether moderators contribute to causal chains; that is, whether they *mediate* associations between abortion and mental health outcomes.

Our topic seems to meet three of the four criteria outlined by Bendersky et al. (2022) for conducting and maintaining a living systematic review (LSR): (1) the topic is relevant for policy and health decision making, (2) there is considerable uncertainty about the evidence for important outcomes, and (3) new evidence is likely to impact conclusions (also see Elliott et al., 2014, 2017). The fourth criterion—availability of sufficient resources to support a LSR—is unknown at this time. We will seek funding to support this systematic review and meta‐analysis, along with important LSR components, if possible.

## 2 OBJECTIVES

We will identify, critically appraise, and synthesize aggregate data from studies of associations between abortion and subsequent mental health outcomes to address the following objectives:

- Assess methodological qualities of studies that assess mental health outcomes of abortion, using a risk of bias framework.
  o To what extent do studies establish time order in associations between abortion and mental health outcomes?
  o To what extent do studies use design or statistical controls for mental health history?
  o To what extent do studies use design or statistical controls for other confounding variables?
- Does abortion increase the risk of mental health outcomes?
  o Is abortion associated with subsequent mental health symptoms or disorders and, if so, which ones?
  o If associations are detected, do they persist after known confounding factors are taken into account?
  o If detected, at what point(s) in time after abortion are associations with mental disorders evident (i.e., are there short‐term, medium‐term, and/or long‐term associations)?
  o If detected, are associations homogeneous or heterogeneous across studies?
- To what extent are associations between abortion and subsequent mental health outcomes moderated by the following study‐level variables:
  o Ability to establish time order in associations;
  o Statistical or design controls for mental health history;
  o Statistical or design controls for other confounding variables;
  o Other risk of bias (RoB) variables (e.g., selective reporting of outcomes);
  o Characteristics of the comparison group (e.g., do results differ depending on whether the comparison group includes a population of all persons capable of pregnancy, or pregnant persons, or people with unintended pregnancies, or people who sought but were denied abortions?);
  o Gestational duration at the time of abortion (first trimester or later); and
  o Characteristics of the geopolitical context (country, state, or province) at the time of the study (e.g., legal restrictions on abortion, country income level).

If sufficient resources are available, we will develop semi‐automated search and screening procedures and workflows needed to create an LSR structure, so that results of this review can be easily updated as new information becomes available.

## 3 METHODS

### 3.1 Criteria for considering studies for this review

Eligible studies must (1) include parallel (non‐overlapping) abortion and comparison groups and (2) obtain data on participants' mental health at least 1 day after the index abortion or after the end of the pregnancy (post‐birth or post‐pregnancy loss) or at a similar point in time on the calendar for cases in the comparison group. Eligibility criteria are described in greater detail below and are summarized in Supporting Information: Appendix 2.

#### 3.1.1 Types of studies

Eligible studies must include at least two parallel groups, including at least one group of people who had abortions and at least one non‐overlapping comparison group (a reference group or control group).

Parallel groups must be non‐overlapping at the time of observation (data collection). That is, for purposes of the study, an individual can only be a member of one group (the abortion group or comparison group) at any point in time, even though her reproductive status may change over time.

We expect some included studies to treat abortion as a status variable that is fixed at a certain point in time; this status is often used to identify an index abortion (if applicable) and determine membership in the abortion group or the comparison group. In these studies, a change in reproductive status (e.g., subsequent pregnancy, abortion, or live birth) should not change participants' initial group membership (just as group member does not change in an intent‐to‐treat analysis, regardless of receipt or completion of the services to which the participants were assigned). We will deal with deviations from this principle as a risk of bias issue (see “avoidance of selection bias, other criteria” below), not as a study exclusion criterion.

We will also include studies that treat abortion as a time‐varying exposure (or status). This approach appears in some epidemiological studies, in which subsequent pregnancies, births, and abortions may be considered. The strengths of this approach often include its reliance on prospective longitudinal data with multiple pre‐ and post‐measures of dependent variables (e.g., mental health).

To ensure that region‐specific regulations related to abortion applied equally to both groups, we will exclude studies that compared historical cohorts (groups that were not observed at the same points in time) as well as studies where the abortion and comparison groups were located in separate jurisdictions (different states, provinces, or countries). We will include multi‐site studies if each jurisdiction contains both abortion and comparison groups.

To avoid the ecological fallacy, we will exclude studies that were not based on individual participant data, such as those that assessed associations between state‐ or place‐based abortion restrictions and aggregate data on mental health outcomes in those areas (e.g., Zandberg et al., 2023).

We will include cross‐sectional, longitudinal, and case‐control studies that meet our eligibility criteria. These three categories include a wide array of study designs; but, because study design labels are not used consistently across disciplines, our criteria rely on key features of eligible studies (as described above), not on study design labels.

#### 3.1.2 Types of participants

Participants include cisgender women, transgender men, and gender non‐binary individuals who are capable of pregnancy and childbirth. Participants may or may not have had previous pregnancies, abortions, and/or live births. There are no restrictions on participants' age.

#### 3.1.3 Types of interventions

We will include abortions that involve the use of certain medications (mifepristone and/or misoprostal, and methotrexate) and/or medical procedures (uterine aspiration, dilation and curettage, dilation and evacuation, or dilation and extraction) that are approved for use in the termination of pregnancies at various stages (gestational durations). These interventions may or may not be legal at the times and/or locations in which they occurred. We will include abortions that occurred at any gestational age.

We assume that most abortions are unwanted pregnancies, most of which are the result of unintended pregnancies although some are also desired pregnancies that are later unwanted due to fetal indications (anomalies), health indications, or due to changes in social or other circumstances (Finer & Zolna, 2011).

Abortions may be self‐reported or identified through any other means (e.g., hospital or administrative records). In studies that recruit patients outside of an abortion clinic setting, self‐reported abortions are typically under‐reported.

We will include self‐managed medication abortions, which may or may not include some assistance from health professionals. We will not include other types of self‐managed abortion or other nonmedical attempts to terminate pregnancy (such as the use of herbs, other substances, or physical methods).

We will exclude studies that focused on multifetal pregnancy reduction, where the aim is to reduce the number of implanted embryos.

#### 3.1.4 Types of comparisons

Because abortion is often legally restricted, care must be taken to ensure that comparison groups are truly comparable to abortion groups to avoid spurious associations and rule out other plausible explanations for associations between abortion and subsequent mental health outcomes. However, there is no consensus about the ideal comparison group in this field. Some observers suggest that ideal comparison cases are people with unintended pregnancies who sought but did not have abortions, but much can be learned from different types of comparison groups. If, for example, mental health outcomes following an abortion are similar to those in the general population, then it is difficult to conclude that abortion increases the risk of mental health problems (especially when population‐based studies provide data on mental health variables at multiple points in time).

Therefore, we will accept many types of comparison groups, but will make distinctions between subgroups of studies in terms of the strength of their counterfactual models, ability to support inferences about associations between abortion and subsequent mental health outcomes, and ability to rule out other plausible explanation for any between‐group differences in outcomes (Shadish et al., 2002). To do this, we will assess risks of bias related to the comparability of abortion and comparison groups (e.g., use of similar inclusion and exclusion criteria, extent to which studies controlled for baseline differences between groups, potential confounding conditions, and differential attrition) in included studies.

Eligible studies include the following types of comparison groups:

- people who could become pregnant,
- pregnant persons (including those with intended pregnancies),
- people with unintended pregnancies, or
- people with pregnancies who sought but did not receive abortions.

We will not include studies that compared people who had medication abortions with those who had procedural abortions, unless the study also includes a non‐abortion comparison group. Relevant comparisons will be limited to abortion versus non‐abortion groups.

#### 3.1.5 Types of outcome measures

Eligible studies must have obtained data on measures of mental health; that is, data on participants' mental health at least 1 day after the abortion, or after the end of pregnancy, or at a similar point in time on the calendar for people who did not have abortions or pregnancies.

We will include studies with and without earlier (e.g., pre‐pregnancy or pre‐abortion) mental health measures. We will handle variations in the availability and use of initial mental health measures as a risk of bias issue, not an eligibility criterion.

We will include studies that used lifetime measures, other incidence and prevalence measures, and contemporaneous measures of mental health outcomes.

We will only include outcome measures that were defined and collected in identical ways (at the same points in time, with identical measurement instruments and data collection procedures) from the abortion and comparison groups. We will have more confidence in outcomes that were assessed with measurement instruments that have evidence of reliability and validity (e.g., Cronbach's alpha or Cohen's kappa > 0.7) in the study sample or in similar samples, but will handle this as a risk of bias issue, not an inclusion criterion.

To address concerns about outcome reporting bias, we will include studies that *measured* one or more primary or secondary outcomes regardless of whether they *reported* results for these outcomes.

##### Primary outcomes

Primary indicators of mental health morbidity are (1) diagnosable conditions (mental disorders, as described in the DSM‐5‐TR or the ICD‐10, or in previous versions of these classification systems), (2) measurable symptoms, (3) reported use of psychotropic medications, and (4) service utilization (outpatient visits, inpatient psychiatric hospitalization) for the following mental disorders that have been linked (in theory and/or past research) to abortion:

- PTSSs or PTSD,
- other anxiety symptoms and disorders,
- depression and other mood disorders,
- suicidal ideation, suicide attempts, and suicide,
- substance use and substance use disorders,
- indicators of any mental disorder, and
- overall mental health symptoms (e.g., scores on the Symptom CheckList [SCL‐90‐R], Derogatis, 1992).

These outcomes may be measured on continuous scales or as dichotomous variables (e.g., the proportion of participants who meet diagnostic criteria for mental disorders).

##### Secondary outcomes

Secondary outcomes include indicators of mental health morbidity (diagnosable conditions, measurable symptoms, reported use of psychotropic medications, outpatient treatments, and psychiatric hospitalization) for the following mental disorders that have more tenuous theoretical and empirical links to abortion:

- eating disorders,
- sleep disorders,
- sexual disorders, and
- psychotic disorders.

As above, secondary outcomes may include dichotomous and continuous measures.

#### 3.1.6 Duration of follow‐up

Although included studies must have at least one set of outcome measures obtained after the end of the index pregnancy (or at a comparable point in time for comparison cases that did not involve pregnancy), we will include all available follow‐up measures in our analyses. We expect the strength of associations between abortion and outcomes to decline over time.

#### 3.1.7 Types of settings

There are no geographic, language, timeframe, or publication restrictions. We will use translation software with any potentially relevant non‐English language materials to ensure that at least two reviewers can independently assess each document and we will rely on our team members' language expertise as appropriate (one member of our review team speaks Danish and one speaks Spanish).

### 3.2 Search methods for identification of studies

The search methods include a variety of strategies designed to maximize the likelihood of finding potentially eligible studies (sensitivity) regardless of their publication status (Kugley et al., 2017; Lefebvre et al., 2019). We will carefully document search strategies so that they can be easily replicated and updated.

#### 3.2.1 Electronic searches

An initial strategy for Medline (Ovid) was adapted from the National Collaborating Centre for Mental Health (NCCMH) (2011) review and further modified based on this review's eligibility criteria and term harvesting. The search was tested against a set of 91 benchmark studies compiled from previous related systematic reviews and a search of Google Scholar, and was peer‐reviewed by three information specialists with experience in evidence synthesis in health and psychology. The complete details of the search strategy development process can be found in Open Science Framework (https://osf.io/v8t92/). The full search strategy for Medline is in Supporting Information: Appendix 1. No date or language limits will be imposed on the search.

A variety of databases containing research in health, psychology, and social sciences, as well as a number of multi‐disciplinary databases and databases with different geographic coverage were selected with the aim of being comprehensive and minimizing bias. The search will be adapted and run in these databases and the exact search strategies, search dates and numbers of results will be documented and reported.

- Medline (Ovid)
- Embase (Ovid)
- Global Health (Ovid)
- CINAHL (EBSCO)
- APA PsycInfo (EBSCO)
- Scopus (Elsevier)
- Social Science Citation Index (SSCI) (Web of Science)
- Science Citation Index Expanded (SCI‐Expanded) (Web of Science)
- Conference Proceedings Citation Index‐Science (CPCI‐S) (Web of Science)
- Conference Proceedings Citation Index‐Social Science & Humanities (CPCI‐SSH) (Web of Science)
- Emerging Sources Citation Index (ESCI) (Web of Science)
- Applied Social Sciences Index and Abstracts (ASSIA) (ProQuest)
- International Bibliography of Social Sciences (IBSS) (ProQuest)
- Sociological Abstracts (including Social Services Abstracts) (ProQuest)
- GenderWatch (ProQuest)
- Latin American and Caribbean Health Science Literature (LILACS): https://lilacs.bvsalud.org/en/
- Africa‐Wide Information (EBSCO)
- Netherland Research Portal, https://netherlands.openaire.eu/
- Research Portal Denmark, https://local.forskningsportal.dk/search/50274
- Cochrane Database of Systematic Reviews (Cochrane Library)
- Epistemonikos: https://www.epistemonikos.org

In addition, we will search databases of theses and dissertations, clinical trials, working papers, and preprints:

- Dissertations and Theses Global (Proquest)
- EThOS e‐theses Online Service: https://ethos.bl.uk/
- Australian Theses and Dissertations: https://trove.nla.gov.au/landing/explore
- The Cochrane Central Register of Controlled Trials (CENTRAL) (Cochrane Library)
- *WHO International Clinical Trials Registry Platform (ICTRP)*: https://www.who.int/clinical-trials-registry-platform
- medRxiv: https://www.medrxiv.org/
- SSRN https://www.ssrn.com/index.cfm/en/
- IDEAS/RePEc: https://ideas.repec.org/

#### 3.2.2 Searching other resources

To identify other potentially eligible studies, we will search relevant gray literature sources (Kugley et al., 2017). A list of websites to be searched is in Supporting Information: Appendix 1.

The following journals and conferences proceedings will be hand‐searched by reviewing the tables of contents for the last 6 months of journal issues or most recent years of proceedings:

- C*ontraception*: https://www.contraceptionjournal.org/
- International Conference on Family Planning: https://icfp2022.org/
- *Population Association of America*: https://www.populationassociation.org/events-publications/future-past-meetings
- *Psychosocial Workshop*: https://www.populationassociation.org/events/event-description?CalendarEventKey=17901441-72e6-479d-9c37-0185f1add416
- Society for Family Planning: https://societyfp.org/learning/annual-meeting/
- Abortion and Reproductive Justice Conference: https://arjc2024.asap-asia.org/

The references of studies included in the review after full‐text screening will be manually screened and relevant references (i.e., potentially eligible studies) will be harvested. Citing references (i.e., forward citations) will be identified using a combination of citation databases (e.g., Google Scholar, Web of Science). The references of other reviews of studies of abortion and mental health (including those cited in Section 1 and in Table 1) will be scanned for additional relevant studies.

We will use a form of snowball sampling to identify researchers and other key informants who are likely to know of relevant unpublished or ongoing studies. We will ask each informant to identify such studies and to identify others who might be able to so. We will continue to contact key informants until no new recommendations are provided. This process will also include calls for studies to listservs and newsletters (Society for Family Planning, WHO Implementing Best Practices Network [IBP], and International Campaign for Women's Right to Safe Abortion Newsletter). Additional information on potentially eligible studies will be sought from study authors as needed.

Google Scholar will also be searched and a set number of results screened for relevance.

### 3.3 Data collection and analysis

#### 3.3.1 Description of methods used in primary research

A variety of methods have been used to assess potential associations between abortion and subsequent mental health outcomes. Studies have used in‐person interviews and other survey methods—which vary in depth and detail—to obtain retrospective and/or contemporary self‐reports on pregnancy intentions, abortions, and other pregnancy events, as well as past and current mental health status and symptoms. Some studies have relied on secondary analysis of survey data, medical records, or national registries. A few examples of the diverse methods employed in this field of research are provided below.

*The Turnaway Study* was a unique, prospective, longitudinal study that examined mental health, physical health, and socioeconomic consequences of abortion compared to being denied an abortion and carrying the unwanted pregnancy to term (Biggs et al., 2015, 2016, 2017; Dobkin et al., 2014; Foster, 2020). From 2008 to 2010, participants were recruited after they requested an abortion in 1 of 30 abortion facilities in the USA. Some participants received abortions because they were under the gestational limit of the clinic, while others were “turned away” and carried the pregnancy to term because they were past the gestational limit. Research assistants interviewed participants by phone every 6 months over a period of 5 years, ending in January 2016. Of the 1132 people who consented to participate, 956 (85%) were interviewed (research participation rates were somewhat lower (79%) in the Turnaway group than in the abortion groups; Dobkin et al., 2014). The interviews covered a wide range of topics including mental health, physical health, employment, educational attainment, relationship status, contraceptive use, and emotions about pregnancy and abortion (Biggs et al., 2017; Foster, 2020).

*Other comparison groups studies with primary data*. Reardon and Ney (2000) mailed a reproductive history questionnaire to homes of a large sample of U.S. women. Analysis was restricted to White women who reported having had one or more abortions (*n* = 137) or no abortions (*n* = 395), and yes/no answers to the question, “Have you ever abused drugs or alcohol?” Broen et al. (2005) followed two groups of Norwegian women from 10 days to 5 years after a first trimester abortion (*n* = 80) or early miscarriage (*n* = 40), and compared results on anxiety, depression, and subjective well‐being at multiple points in time while controlling for potential confounders. Gilchrist et al. (1995) conducted a large, prospective, longitudinal study of women with unplanned pregnancies in the UK (6410 had abortions, 6151 did not seek abortions, 379 sought and were denied abortions, and 321 sought abortions but changed their minds). Post‐delivery/abortion psychiatric outcomes included psychosis, nonpsychotic illness, deliberate self‐harm without other psychiatric illness (e.g., drug overdose), and no psychiatric illness. Between‐group comparisons controlled for measures of prior mental health as well as other covariates (e.g., age, marital status, and prior history of abortion).

*Secondary analysis of survey data*. Relevant studies have been based on secondary analysis of national longitudinal data sets compiled in the USA, such as the National Longitudinal Survey of Youth (NLSY), the Health of American Women Survey (HAWS), the National Pregnancy and Health Survey (NPHS), National Longitudinal Study of Adolescent Health (Add Health), National Survey of Family Growth (NSFG), National CoMorbidity Survey (NCS), and on data sets from specific U.S. metropolitan areas (Major et al., 2009). These data sets provide opportunities for different teams of researchers to review and replicate or challenge the work of others (see, e.g., Steinberg & Finer, 2011; Steinberg et al., 2014).

*Secondary analysis of medical records*. Several papers by Reardon and colleagues were based on overlapping samples of medical records from California's state‐funded medical insurance program for low‐income families. These studies excluded women who had subsequent abortions from the delivery group, but not from the abortion group (Major et al., 2009).

*Secondary analysis of national registries*. Munk‐Olsen and colleagues conducted a population‐based cohort study, by linking information from three Danish national registries from 1995 to 2007 to obtain data on a sample of girls and women who had no record of mental disorders when they had either a first‐trimester induced abortion or a first childbirth. They estimated rates of first‐time psychiatric contact (inpatient admission or outpatient visit) for any type of mental disorder within 12 months after the abortion or childbirth, compared with the 9‐month period preceding the event (Munk‐Olsen et al., 2011). Other studies have been based on national registry data in Finland (Major et al., 2009).

#### 3.3.2 Selection of studies

As described below, the selection of studies for our review will occur in two stages: (1) screening of titles and abstracts and (2) eligibility decisions based on full text reading of all relevant study reports.

Search results (titles and abstracts) will be imported into Covidence for screening purposes. For titles that lack English language abstracts, we will retrieve and/or translate the abstract (or a portion of the document, if needed), and will upload this information into Covidence before screening. We will use Google translate to produce English versions of non‐English abstracts and documents.

We will use the machine learning (ML) tool within Covidence to facilitate screening (Chappell et al., 2023). Informed by human screening decisions, Covidence uses machine ranking and continuous machine training to sort citations in the order of relevance.

Working independently, two reviewers will apply the screening tool (shown in Supporting Information: Appendix 2, Stage 1) to titles and abstracts. Screening decisions are Yes (potentially eligible), No (not eligible), and Maybe. We will sort these records into three groups: both reviewers voted Yes, both voted No, and conflicting opinions or Maybe votes. We will meet periodically to discuss conflicting opinions and resolve those that are clearly Yes or clearly No, leaving unresolved records as is until the end of screening.

Using ML to sort records by relevance, we will use a stopping rule to transition from two screeners per record to one screener as we approach less relevant records. Only one reviewer will screen the remaining records, which are expected to be not eligible or unclear. As the use of ML for this purpose is rapidly evolving, we will consult with experts on the pros and cons of various stopping rules and will select a stopping rule that is transparent, sensitive, and efficient.

At the end of screening, unresolved records will be promoted along with Yes (potentially eligible) records for full text retrieval.

Before study eligibility decisions are made, citations will be collated to determine which citations (reports) are associated with which unique studies. A unique study involves a sample that does not overlap with another study sample in this review. Studies may have multiple citations and citations may include information on multiple studies (non‐overlapping samples).

Study eligibility decisions will be made by two reviewers working independently, using the second half (Stage 2) of the screening and eligibility tool shown in Supporting Information: Appendix 2. Reviewers will compare notes after they have completed their assessments and will discuss and attempt to resolve any discrepancies. Discrepancies that are not resolved by the first two reviewers will be discussed and resolved with a third reviewer. Reasons for exclusion will be documented for all studies excluded at this stage (based on full text analysis) and a full list of these excluded studies with specific reasons for their exclusion will be provided in the final review.

#### 3.3.3 Data extraction and management

For each eligible study, data extraction will be conducted by at least two review team members who were not involved in the conduct of that study. Working independently, two reviewers will extract data using the form shown in Supporting Information: Appendix 3. As above, when finished with their independent assessments, reviewers will compare notes, resolve discrepancies, and consult with a third reviewer when necessary to resolve discrepancies.

For each study, we will extract information on:

- Study or data set characteristics;
- Sub‐study characteristics (to distinguish multiple analyses derived from shared data sets, such as the U.S. National Comorbidity Study): comparison groups, study design, timing, locations;
- Participant characteristics: demographics, socioeconomic characteristics, initial mental health status;
- Measurement tools: reliability and validity of outcome measures;
- Effect size data: raw data, valid subgroup Ns, attrition, analytic models, covariates (e.g., statistical controls for initial differences between groups); and
- Study‐level risks of bias.

As shown in Supporting Information: Appendix 3, data extraction will be structured hierarchically, with up to five nested levels: study‐level data, sub‐study‐level data, report‐level data, measurement‐level data, and effect‐size‐level data. For purposes of data analysis, we will generate an effect‐size‐level file (a spreadsheet) with all relevant study‐, sub‐study‐, report‐, and measurement‐level data for each unique effect size (ES). This structure will handle repeated measures, by treating them as separate (unique) ES.

We will try to obtain data on the nature of any legal restrictions on abortion that were in place at the time and location of each included study. If this information is not provided in the study reports, we will search the Center for Reproductive Rights (2023) database on the world's abortion laws for information relevant for included studies (https://reproductiverights.org/maps/worlds-abortion-laws/).

Meta‐Reviewer (https://www.metareviewer.org/) will be used for data extraction because it is designed to support duplicate extraction and a hierarchical data structure. Online data extraction forms will mirror the forms shown in Supporting Information: Appendix 3 and will be used to store all (duplicate) coding decisions, along with all resolutions (of initial coding differences) and validation checks.

All coders will independently pilot test the screening and data extraction forms and revisions will be made as needed to improve the clarity, efficiency, and inter‐rater reliability of these tools. We will apply all criteria, questions, and coding uniformly across all included studies.

#### 3.3.4 Assessment of risk of bias in included studies

Included studies will be judged against the following criteria, adapted from the Cochrane Risk of Bias tool (version 1.0, Higgins & Green, 2011), the What Works Clearinghouse standards for initial group equivalence and attrition (What Works Clearinghouse, 2018a, 2018b), previous Campbell systematic reviews (Littell et al., 2005, 2008, 2021, 2023; Valentine et al., 2023), and the confounder matrix for assessing observational studies of etiology (Petersen et al., 2022). Risk of bias ratings will not be used as criteria for inclusion in the synthesis but may be used in subgroup and moderator analyses.^1^

*Time order*: study design establishes a clear temporal order for abortion and mental health measures.

- Yes = Low risk: longitudinal study collected data on mental health measures at specific time points/intervals (e.g., present time, within last 3 months, within last 12 months) before and/or after abortion or other pregnancy events (or at similar points in time on the calendar for comparison cases).
- Unclear risk = insufficient information.
- No = High risk: use of correlational data, lifetime measures of abortion, and/or lifetime measures of mental health outcomes.

*Avoidance of confounding (initial equivalence on mental health history)*: initial differences between groups on measures of mental health history were small or moderate (*d* ≤ 0.25) or researchers used statistical controls (e.g., propensity score matching, regression covariates) for initial differences.

- Yes = Low risk
- Unclear risk: insufficient information (e.g., group‐level data were not provided, *d* cannot be computed, unclear if statistical controls were sufficient to create comparable groups)
- No = High risk: there were initial differences between groups with *d* > 0.25, no/inadequate statistical controls for these differences, or no measures of mental health history.

*Avoidance of confounding (pregnancy intention)*: comparison groups were matched on pregnancy intention (e.g., pregnancy intention was used as an inclusion criterion, used in matching designs and/or the proportion of cases with unintended or unwanted pregnancies was equivalent (*d* ≤ 0.25) across groups).

- Yes = Low risk.
- Unclear risk = insufficient information.
- No = High risk: between group differences on pregnancy intentions (*d* > 0.25).

*Avoidance of confounding (initial equivalence on background characteristics)*: initial differences between groups on socioeconomic variables (e.g., income, education, employment) and family background characteristics (e.g., adverse childhood experiences, interpersonal violence) were small or moderate (*d* ≤ 0.25) or researchers used statistical controls (e.g., propensity score matching, regression covariates) for baseline differences.

- Yes = Low risk
- Unclear risk: insufficient information (e.g., group‐level data were not provided, *d* cannot be computed, unclear if statistical controls were sufficient to create comparable groups)
- No = High risk: there were initial differences between groups with *d* > 0.25, and no/inadequate statistical controls for these differences.

*Avoidance of confounding (other criteria)*: comparison groups were constructed and maintained with similar inclusion and exclusion criteria (e.g., related to age, prior pregnancies, live births, abortions, and prior mental health status), taking into account any country‐ or region‐specific regulations limiting access to abortion to certain groups.

- Yes = Low risk.
- Unclear risk = insufficient information.
- No = High risk: inclusion or exclusion criteria were applied differently to different comparison groups.

*Avoidance of confounding (performance bias)*: there were no systematic differences (*d* ≤ 0.25) between groups (or use of statistical controls for differences between groups) in terms of levels of service, care, attention, perceived stigma, or social support.

- Yes = Low risk
- Unclear (insufficient information)
- No = High risk: one group received more services, care, attention, perceived stigma, or support and these factors were not accounted for in the analysis.

*Avoidance of detection bias* (blinding): assessor is unaware of group membership when collecting outcome data.

- Yes for all outcomes = Low risk
- Yes for some outcomes = Unclear
- Unclear (insufficient information)
- No = High risk

*Avoidance of attrition bias*: Losses to follow up were less than or equal to 25% and equally distributed (≤10% difference in response rates) across groups. Group equivalence on important initial characteristics was retained after losses to follow‐up (*d* ≤ 0.25).

- Yes for all outcomes = Low risk
- Yes for some outcomes = Unclear
- Unclear (insufficient information)
- No = High risk: loss of initial equivalence (*d* > 0.25), losses to follow up >25% overall, or losses were unequally distributed (>10% difference) across groups.

*Standardized observation periods*: follow‐up data were collected from each case at a fixed point in time (e.g., after the end of pregnancy), or analyses included statistical controls for variable observation periods (e.g., event history analysis).

- Yes for all outcomes = Low risk
- Yes for some outcomes = Unclear
- Unclear (insufficient information)
- No = High risk

*Validated measures of mental health history*: use of instruments with demonstrated reliability (e.g., alpha/kappa > 0.7) or validity in the study sample or in similar samples (from similar countries, socioeconomic and racial/ethnic groups), or use of administrative data on mental health events (e.g., prior psychiatric hospitalizations).

- Yes = Low risk: reliable mental health history measures were collected before, during, or within 14 days after pregnancy and at the same point(s) in time for all groups.
- Unclear risk = insufficient information on reliability, validity, or timing of baseline mental health measures.
- No = High risk: reliance on retrospective reporting, single item self‐reports, or unreliable measures (alpha/kappa < 0.7) or no measures of mental health history.

*Validated mental health outcome measures*: use of instruments with demonstrated reliability (e.g., alpha/kappa > 0.7) or validity in the study sample and/or similar samples from (similar countries, socioeconomic, and racial/ethnic groups), or use of external administrative data on events (e.g., psychiatric hospitalization).

- Yes for all outcomes = Low risk
- Yes for some outcomes = Unclear
- Unclear (insufficient information)
- No = High risk

*Free of selective reporting*: a prospective study protocol is available and all pre‐specified outcomes are reported in the pre‐specified way; all expected outcomes are reported in full and for all cases (e.g., no systematic exclusion of subgroups of cases), regardless of the direction and statistical significance of results.

- Yes = Low risk
- Unclear (e.g., prospective protocol is not available, or changes in the protocol were made after the study began)
- No = High risk: some outcomes are not reported or are reported incompletely (e.g., non‐significant results are mentioned, but data are not provided; data are provided for selected subgroups only).

All risk of bias assessments will be documented in a section on Characteristics of Included Studies, with support for each judgment, including verbatim passages from study reports with clear citations. Results will be summarized in a Risk of Bias Graph and a Risk of Bias Summary with data on each rating for all included studies (e.g., see Littell et al., 2021, 2023).

#### 3.3.5 Measures of treatment effect

Certain ES metrics will be used to compare results obtained from two parallel groups (an abortion and comparison group) on continuous and dichotomous outcome measures.

*Continuous outcomes*. Between‐group differences on continuous outcome measures, such as scores on standardized measures of symptoms of mental disorders, will be analyzed using Hedges' *g*, the small‐sample adjusted standardized mean difference (SMD) between the two groups. We will follow the US What Works Clearinghouse (WWC, 2022) guidelines for computing Hedges' *g* using unstandardized regression coefficients and the pooled, unadjusted sample standard deviations (see appendix E in WWC, 2022). If only unadjusted summary statistics (means and standard deviations, *t*‐tests, etc.) are reported, we will extract these estimates to compute Hedges' *g*, but will treat unadjusted estimates separately from covariate‐adjusted estimates in the analysis (as described below).

*Dichotomous outcomes*. Studies may report the odds or risks of a mental health condition in the two groups or the time‐to‐event/hazards ratios for both groups. Risk ratios will be converted to odds ratios (ORs), following the guidance in Borenstein and Hedges (2019), using the risk of the event in the comparison group as the baseline risk. For purposes of analyses, ORs and their associated standard errors will be converted to log units (LORs) and their associated standard errors. For studies that use logistic regression models to estimate a covariate‐adjusted OR, we will extract the adjusted OR and its associated SE, and then convert these statistics to the log‐odds ratio (LOR) and its associated SE for purposes of meta‐analysis. Then, LORs and their SE will be converted back to ORs and their SEs for purposes of reporting and interpretation.

Studies may report hazard ratios for the odds of a particular outcome occurring during a certain time period. We will extract hazard ratios if no other information is available. We will explore the use of methods Watkins and Bennett (2018) provided for converting binomial counts/proportions from two groups allowing the synthesis of studies reporting either hazard ratios or binomial counts/proportions.

*Adjusted and unadjusted effect sizes*. For both continuous and dichotomous outcomes, effect sizes that are adjusted for initial between‐group differences will be prioritized and treated separately from unadjusted estimates in the meta‐analysis models. Studies may present the results of multiple statistical models that control for various differences between the two groups. We will prioritize effect sizes that adjust for the following initial between‐group differences:

- mental health history,
- age,
- exposure to violence or trauma,
- socioeconomic status (income, education, poverty status),
- marital or relationship status, and
- number of children.

As we extract data, we will use coding to capture the covariates used in each model (for each adjusted ES). If studies only present unadjusted ES, we will extract these estimates, but will treat them separately in the meta‐analysis (see Section 3.4 below).

When reports provide insufficient data for effect size calculations, we will attempt to retrieve additional information from the study authors.

After computing effect sizes, we will examine outliers and check to make sure that our data accurately reflect study reports.

#### 3.3.6 Unit of analysis issues

It is possible that some participants will have multiple pregnancies (events) during the study time period. Abortion and comparison groups are defined by the conclusion of an index pregnancy (usually the first pregnancy that occurs in the study timeframe) or the passage of a fixed amount of time. We assume that included studies will treat individual participants (not pregnancies or abortions) as the primary unit of analysis (i.e., an individual should not be double‐counted if they had two pregnancy‐related events).

Studies with more than two comparison groups may provide useful opportunities to examine multiple contrasts (e.g., abortions vs. live births and abortions vs. miscarriages). We will extract all relevant data from all eligible comparison groups, taking care to keep these estimates separate in pairwise meta‐analysis (data on one group cannot be used twice in the same pairwise meta‐analysis). As explained below, all outcome data will be included in correlated and hierarchical effects (CHE) models, but different contrasts will be coded as such.

Similarly, we expect to find multiple analyses (and multiple reports) based on shared data sets. For purposes of our review, a data set is a unique sample of participants that does not overlap with another data set; thus, a data set is considered a “study” in our review. Sub‐studies are based on shared data sets, but may use different comparison groups (i.e., different inclusion and exclusion criteria), different time frames, covariates, and outcome variables. We will assess the overlap between sub‐studies that relied upon the same data sets to determine if they share participants; methods for handling overlapping samples and other sources of dependencies among effect sizes are described in the next section.

Multiple measures of the same outcome (e.g., multiple scales and/or dichotomous measures) will be included in the analysis, following plans detailed in the Data Analysis section.

If studies use clustered samples and did not adjust for clustering, we will adjust the standard errors of the SMDs using the methods described in the WWC 5.0 Procedures and Standards Handbook (What Works Clearinghouse, 2022). For ORs reported from unadjusted clustered samples, we will adjust the standard error of the LOR using methods recommended in the Cochrane Handbook.

#### 3.3.7 Criteria for determination of independent findings

We will include data from all available study reports on all relevant outcomes, contrasts, and endpoints in our analysis. We are interested in mean effects and in exploring the heterogeneity of effects across studies (i.e., both convergent and divergent approaches), so we will deal with effect size multiplicity (multiple statistically dependent effect sizes) by using both reductionist and integrative meta‐analytic techniques (López‐López et al., 2018). As described below, we will conduct two separate types of synthesis: pairwise meta‐analysis (using a reductionist approach) and CHE models (an integrative approach).

We will construct as many pairwise meta‐analyses as are needed to capture conceptually distinct outcomes and distinct time intervals. To avoid multiplicity, each study can contribute only one effect size to each pairwise meta‐analysis. We will select one ES per study for each pairwise meta‐analysis, using the following selection rules.

We will collapse endpoints into common non‐overlapping intervals, for example: less than 90 days after pregnancy, 3–6 months, 7–11 months, and 12–23 months, 24–35 months, 3–5 years, and so forth. If a study has more than one relevant data point within a particular time interval, we will use the latest endpoint within that interval in that pairwise meta‐analysis.

When studies report multiple measures of the same outcome (e.g., depression) at the same point in time, we will select the most comprehensive measure (e.g., overall depression score) over single items or single/selected symptoms for pairwise meta‐analysis. If outcome data are provided by multiple sources (self‐reports, collateral interviews, and official records), we will use the most direct or reliable measure in pairwise meta‐analysis; self‐reports on validated measures are considered more direct than official records, and collateral reports are considered less direct that self‐reports and official records.

Data from multiple sub‐studies or multiple reports with overlapping samples will not be included in the same pairwise meta‐analysis to avoid duplication of cases and dependent effect sizes. Instead, we will display results from overlapping samples as separate subgroups in forest plots to show results of different analyses based on the same (or overlapping) sample of cases; results will not be pooled (averaged) across subgroups with overlapping samples.

We will use CHE models with robust variance estimates (RVE) to combine multiple dependent effect sizes across studies (Pustejovsky & Tipton, 2022). Described below (in Section 3.4), these analyses allow us to include multiple effect sizes per study (including multiple measures of the same outcome, multiple endpoints, multiple subgroups, and multiple comparisons) in statistical models that adjust for multiplicity.

#### 3.3.8 Dealing with missing data

We will request missing data from the primary authors of included studies when needed to understand study characteristics, complete risk‐of‐bias assessments, or calculate effect sizes, paying particular attention to outcome data that were collected but not reported or under‐reported.

We will record data on attrition and differential attrition for each effect size (each outcome and each endpoint). If there is missing data for more than 50% of one or both comparison groups, we will not include that effect size in meta‐analysis.

Where possible, we will use Cochrane's revman‐calculator to calculate missing standard deviations.

We expect eligible studies to differ in the reporting of moderators that we plan to use in exploring heterogeneity. Before using statistical methods to explore the sensitivity of results to missing covariates, we will conduct and report an exploratory analysis of the amount and patterns of missing data as described by Schauer et al. (2022). These exploratory methods can highlight what covariates are incompletely reported as well as patterns of incomplete data across studies. Research is ongoing regarding the application of standard missing data methods such as multiple imputation to meta‐analysis (see e.g., Lee & Beretvas, 2023; Schauer, 2019). A concern raised by this research is the association between effect size and likelihood of missing values, a situation unique to meta‐analysis. We will consider the use of multiple imputation as a method of checking the sensitivity of our results to missing data depending on the pattern and prevalence of missing covariates.

#### 3.3.9 Assessment of heterogeneity

We will evaluate heterogeneity with the Chi‐square test of heterogeneity, *I* ^2^ (in pairwise meta‐analysis), and tau‐squared (*τ* ^2^). For CHE models estimating the mean effect size, we will present the square‐root of the within‐study $\left(\omega^{2}\right)$ and between‐study components of variance (*τ* ^2^), and the prediction interval. We will also consider presenting the multivariate version of *I* ^2^ as suggested by Viechtbauer (2022).

With sufficient studies, we will use meta‐regression to explore associations between study and participant characteristics (potential moderators of effects) and the direction and magnitude of an effect size.

#### 3.3.10 Assessment of reporting biases

We will use funnel plots to assess the risk of publication bias and other potential sources of bias. If there are at least 10 studies (independent effect sizes) in a funnel plot, we will use statistical tests for asymmetry (e.g., Egger's test) for dependent effect sizes as discussed by Rodgers and Pustejovsky (2021) and an extension to selection models using cluster wild bootstrapping methods (Pustejovsky & Joshi, 2023). We will also consider the use of other methods for exploring the sensitivity of results to publication/small sample bias such as those developed by Mathur and VanderWeele (2020).

We will use all available reports on included studies (included registered protocols, if available) to track reporting of outcomes within studies, across all endpoints, and by outcome domain. Results of these analyses will be arrayed graphically and used to support judgments regarding risks of selective reporting (e.g., see Littell et al., 2021, 2023).

### 3.4 Data synthesis

We will use pairwise meta‐analysis to synthesize data from multiple, non‐overlapping studies on comparable outcome measures at similar points in time. We will use CHE meta‐analysis models to synthesize data on all available outcomes within conceptually distinct outcome domains. These two different kinds of meta‐analysis are explained below.

We do not expect all studies to produce estimates of the same population parameters (i.e., risks of subsequent mental health problems associated with abortion), given the likely differences between studies in research designs, settings, participant characteristics, and outcome measures. Therefore, we will use random effects meta‐analysis models whenever possible (i.e., in pairwise meta‐analysis and in CHE models with *df* > 4).

#### 3.4.1 Pairwise meta‐analysis

We will conduct separate pairwise meta‐analyses for conceptually distinct outcomes, at specific endpoints, and within the following outcome domains:

- PTSSs or PTSD,
- other anxiety symptoms and disorders,
- depression and other mood symptoms or disorders,
- suicidal ideation and suicide attempts,
- substance use and substance use disorders,
- any mental disorder or mental health symptoms,
- eating disorders,
- sleeping disorders,
- sexual disorders, and
- psychotic disorders.

We plan to present data in subgroups by study design variables, and display results in forest plots alongside study ROB ratings. If possible, contrasts between cases with abortions and live births will be kept separate from contrasts between cases with abortions and other pregnancy outcomes (e.g., miscarriages) or no pregnancy outcome. In some analyses we may compare abortion with all other outcomes (including comparisons with people who were not pregnant).

Pairwise meta‐analysis will be conducted in the R program *metafor* (Viechtbauer, 2010). We will use Hedges' *g*, the small‐sample adjusted SMD for continuous outcomes. Inverse variance methods will be used to pool SMDs, so that each effect size is weighted by the inverse of its variance in an overall estimate of effect size. Confidence intervals (CIs) of 95% will be used for individual study data and for pooled estimates.

#### 3.4.2 CHE models

Observational studies often report multiple dependent outcomes, including multiple measures of the same construct, reports on the same measure from multiple data sources, and repeated measures from the same sources over time. We will use the CHE model described by Pustejovsky and Tipton (2022) to handle these dependencies, and RVE to guard against model mis‐specification. The CHE model assumes that effect sizes are correlated and nested within studies, because they are derived from the same sample or from the same study. This approach provides “valid point estimates, standard errors, and hypothesis tests even when the degree and structure of dependence between effect sizes is unknown” (Fisher & Tipton, 2015, p. 1; also see: Hedges et al., 2010; Tanner‐Smith & Tipton, 2014; Tanner‐Smith et al., 2016).

We will use small sample corrections for RVE with meta‐analysis (Tipton, 2015).

Studies can report similar outcomes in different ways (e.g., days of drug use vs. days of abstinence from drug use). Therefore, before performing the CHE analysis, we will reverse score some outcomes so that higher scores always represent more severe mental health problems.

We will use all available data on primary and secondary outcomes in the CHE models, including multiple measures of the same outcome at different points in time. We will assume a correlation of 0.6 for effect sizes measured within the same study, but will test this assumption with sensitivity analysis, assessing results for rho (*ρ*) = 0.4, 0.6, and 0.8. Results will show whether different values of rho produce consistent estimates of mean ES coefficients, standard errors, and tau‐squared (*τ* ^2^).

We will estimate effect size models (both the mean effect size model and any moderator models) using the R programs *metafor* (Viechtbauer, 2010) and *clubSandwich* (Pustejovsky, 2023).

For dichotomous outcomes, our synthesis will be conducted using the LOR, and we will convert results back to ORs for ease of interpretation. Then, to increase statistical power, we will conduct an analysis combining SMDs and studies reporting dichotomous outcomes. For studies reporting dichotomous outcomes, we will compute the Cox index, an effect size comparable to Hedges' *g* and used by the What Works Clearinghouse (2022). If there are too few studies with valid effect sizes for meaningful analyses within each outcome domain, we will collapse all primary mental health outcomes (the first six domains) and all secondary outcomes (the last four domains) into larger CHE analyses.

Estimates from CHE models with fewer than four degrees of freedom are unreliable (Tanner‐Smith & Tipton, 2014). In these instances (when there are fewer than five studies reporting on an outcome in the analysis), we will examine forest plots of all relevant ES. We will use forest plots that reflect the dependent effect size structure, such as those included in Winters et al. (2022). We will then consider aggregating these ES within studies (computing a study‐level ES) using the aggregate function in metafor and following recommendations by Pustejovsky (2019). We will assume a correlation of 0.6 for associations among effect sizes within studies. We will then use fixed effect (FE) models to estimate the mean ES across studies. We will also consider using cluster wild bootstrapping methods for estimating dependent effect size models with a small number of studies (Joshi et al., 2022).

Where possible, we will provide 95% prediction intervals (PIs) as well as 95% CIs around point estimates of main effects. PIs show the range of values within which results of future studies are likely to fall. Fixed effect models assume that tau‐squared (*τ* ^2^) is zero; thus, there are no PIs for fixed effect models.

#### 3.4.3 Subgroup analysis and investigation of heterogeneity

We plan to fully document and transparently report the decisions we make to explore effect size heterogeneity. We anticipate overlap and multicollinearity among the planned moderators. We will explore these correlations and the distribution of moderator levels both within and across studies. Power for tests of moderators likely relates not only to the number of studies/effect sizes within each subgroup but also their balance across studies/effect sizes.

If available data support moderator analysis (e.g., at least 5 effect sizes per subgroup/moderator level)), we will examine the following variables as potential moderators of associations between abortion and subsequent mental health outcomes:

- Study design characteristics including:
  ∘ establishment of time order (abortion precedes mental health outcomes),
  ∘ design/statistical controls for mental health history and other confounding variables,
  ∘ characteristics of the comparison group, and
  ∘ other risk‐of‐bias ratings;
- Gestational duration at the time of abortion (e.g., first trimester or later); and
- Context at the time and location of the study:
  ∘ type(s) of legal restrictions on abortion (Center for Reproductive Rights, 2023), and
  ∘ country income level (low, middle, high; World Bank, 2023).

We expect to find larger effect sizes (associations) when studies do not clearly establish time order, when ES were not adjusted for mental health history and other known confounding variables, in studies with more dissimilar comparison groups (e.g., where the groups differ in pregnancy intention), and when other risks of bias are present (e.g., lack of initial group comparability). We expect larger ES in studies where a higher proportion of participants had abortions after the first trimester. Finally, we expect larger ES in contexts with more stringent legal restrictions on abortion and in settings where there is less access to health care.

If possible, we will also perform exploratory analyses with the following potential moderators:

- Type of outcome measure (e.g., short standardized self‐report, registry data, in‐depth interview)
- Year(s) of data collection (calendar effects)
- Type of abortion: procedural or medication abortion,
- Lag time between abortion and mental health outcome measures,
- Mean age of pregnant persons,
- Participants' socioeconomic status (income, education, poverty),
- Number of prior live births, and
- Perceived stigma related to abortion.

We will explore how groups of moderators relate to effect size heterogeneity, starting with methodological characteristics of studies, then average gestational duration, context, and (if possible) type of outcome measure and other potential moderators listed above. We will treat these effect size models as exploratory, using caution in interpreting the results. We are likely to have both direct and indirect evidence about effect size heterogeneity as detailed by Pustejovsky and colleagues (2023), and will ensure that results are interpreted appropriately.

If possible, we will estimate ES for specific outcome domains (see Section 3.1.4).

#### 3.4.4 Sensitivity analysis

We will use sensitivity analysis to examine the potentially biasing effects of outliers (e.g., studies with unusually large sample sizes, and those with extremely high or low ES). Sensitivity analyses will be performed by removing studies one at a time from a forest plot or from a CHE analysis and comparing results with and without a study. We will use caution in defining outliers as they are dependent on context. We will start with Tukey's (1977) suggestion to define outliers as effect size estimates falling more than three times the interquartile range below the first quartile or above the third quartile.

As mentioned above, we will assess the sensitivity of CHE and FE models to various assumptions about the size of the correlations between effect sizes within studies.

If our plans change in ways that deviate from this protocol, we will document these changes and report them—along with our rationale for making changes—in the final report (in a section on Deviations from the Protocol). Where possible, we will conduct sensitivity analysis to assess the possible impact of deviations from the protocol on the results.

#### 3.4.5 Treatment of qualitative research

We do not plan to include qualitative research.

#### 3.4.6 Summary of findings and assessment of the certainty of the evidence

We will use the GRADE guidelines (gdt.gradepro.org) to assess the certainty of evidence regarding primary outcomes in a Summary of Findings (SoF) table. If possible, the SoF table will include measures of each of our six primary outcomes at approximately 1 year after pregnancy ends.

### 3.5 Data sharing plans

Data sharing is not applicable to this protocol as no data sets were generated or analyzed for during the development of the protocol.

We will publish the raw data from the completed systematic review and meta‐analysis (i.e., the aggregate data that we extracted from included studies) as a csv file, along with a codebook, and the R syntax used to generate all statistical analyses.

Before publication of the final report on this project, we will seek independent replication of the results of our CHE analysis by a meta‐analyst who is not associated with our review and is not aware of the meaning of the direction of effect sizes (i.e., does not know which groups is favored by positive or negative ES).

If possible, we will introduce crowd‐sourcing and semi‐automation of critical procedures (e.g., searching and screening) and workflows to develop and maintain an LSR (Bendersky et al., 2022; Elliott et al., 2014, 2017).

## CONTRIBUTIONS OF AUTHORS

Julia H. Littell (JHL), Trine Munk‐Olson (TMO), and Julia R. Steinberg (JRS) conceived the idea for this project, along with Chelsea B. Polis. JHL drafted the protocol with Sarah Young (SY) and Therese D. Pigott (TDP). All other co‐authors provided input, reviewed, and commented on multiple drafts of the protocol; these co‐authors (Content experts) are listed in alphabetical order by last name.

Content: M. Antonia Biggs (MAB), TMO, and JRS are experts in the content area.

Systematic review methods: JHL, SY, and TDP are experts in systematic review methods.

Information retrieval: SY is an expert in information retrieval.

Statistical analysis: TDP is an expert in meta‐analysis.

## DECLARATIONS OF INTEREST

MAB, TMO, and JRS have been involved in the conduct and reporting of primary research on the topic. As explained in the Methods section, under Data Extraction and Management, any author of a study considered for potential inclusion in this review will not be involved in the study eligibility decision, data extraction, or coding of that study.

## PRELIMINARY TIMEFRAME

We plan to submit the final review by December 2026.

## PLANS FOR UPDATING THIS REVIEW

The review will be updated by every 5 years or more often, if sufficient resources are available.

## SOURCES OF SUPPORT

Internal sources: None.

External sources: None.

## Supporting information

Supporting information.

## Footnotes

1 Our approach differs from the ROBINS‐I tool which was developed for assessment of risks of bias in nonrandomized studies of intervention effects (Sterne et al., 2016). ROBINS‐I recommends *post hoc* exclusion of studies with a critical risk of bias rating on any one (or more) items. In our view, different types of risk of bias are likely to have different impacts on effect sizes (ES), and these relationships should be explored empirically when possible. Further, the ROBINS‐I approach often leads to *post hoc* exclusion of otherwise‐eligible studies without transparent documentation of reasons for exclusion (specific risks of bias). We think that full critical appraisal of all studies that meet our eligibility criteria is important, given controversies over the interpretations and uses of results of these studies.
