# Correction to “Abortion and mental health outcomes: A systematic review and meta‐analysis”

**DOI:** 10.1002/cl2.1437

**Published:** 2024-09-02

Littell, J. H., Young, S., Pigott, T. D., Biggs, M. A., Munk‐Olsen, T., & Steinberg, J. R. (2024). Abortion and mental health outcomes: A systematic review and meta‐analysis. *Campbell Systematic Reviews*, *20*, e1410. https://doi.org/10.1002/cl2.1410

There was an omission of “Protocol” at the beginning of the title for this protocol. The correct title is as follows:

PROTOCOL: Abortion and mental health outcomes: A systematic review and meta‐analysis

We apologize for this error.

