# Supplementary material for: PROTOCOL: Abortion and mental health outcomes: A systematic review and meta‐analysis
Source: Campbell Syst Rev. 2024 May 21;20(2):e1410. doi: 10.1002/cl2.1410 (PMC11109527; doi:10.1002/cl2.1410)
Supplement: Supplementary file 1 — Supporting information. [file CL2-20-e1410-s001.pdf]

Appendix 1: Search strategy

Search strategy for Medline (Ovid)

1	Abortion, Induced/ OR Abortion, Legal/ OR Abortion, Therapeutic/ OR Abortion, Eugenic/ OR Abortion Applicants/ OR Abortion, Criminal/ OR exp Abortion Seekers/
2	(abort* OR postabort* OR "post-abort*" OR "post abort*" OR feticide OR foeticide OR ((f?etal* OR f?etus* OR gestat* OR interpregnan* OR midtrimester* OR "mid-trimester*" OR pregnan* OR prenatal* OR "pre natal*" OR trimester*) adj3 (terminat* OR "post-terminat*" OR postterminat*)) OR (pregnanc* adj2 resolution) OR (((elective* OR voluntar* OR induced) adj2 (interrupt* OR terminat*)) AND (f?etal* OR f?etus* OR gestat* OR interpregnan* OR midtrimester* OR pregnan* OR prenatal* OR "pre natal*" OR trimester*))).ti,ab,kf,ot,oa.
3	1 OR 2
4	Mental disorders/ OR Mental health/ OR Psychological Well-Being/ OR Psychological Trauma/
5	(((mental* OR psychological* OR psychiatric* OR emotional OR psychotic OR stress) adj2 disorder*) OR ((mental* OR psycho* OR psychiatric*) adj3 (condition* OR disease* OR health OR ill* OR problem* OR well-being OR "well being" OR response* OR distress OR stress OR trauma* OR effect$1)) OR ((psychological* OR psychiatric*) adj (episode* OR outcome* OR morbidity OR consequence* OR sequela* OR symptom* OR implication* OR maladjust* OR indication* OR reaction*)) OR "life satisfaction").ti,ab,kf,ot,oa.
6	exp Mood Disorders/ OR Depression/ OR Affective Symptoms/
7	(depression OR depressive* OR anxiety OR ((mood OR affective) adj disorder) OR "affective symptom*" OR bipolar OR mania* OR manic* OR dysphori* OR dysthymi* OR rapid cycl$ OR hebephreni* OR MDD).ti,ab,kf,ot,oa.
8	exp Anxiety Disorders/ OR Stress Disorders, Post-Traumatic/
9	(anxiet* OR anxious* OR obsessive* OR compulsi* OR ocd OR panic* OR phobi* OR agoraphobi* OR claustrophobi* OR "post- traumatic*" OR posttraumatic* OR PTSD OR "recur* thought*").ti,ab,kf,ot,oa.
10	exp Psychotic Disorders/ OR exp Schizophrenia/ OR Affective Disorders, Psychotic/ OR Delusions/ OR Hallucinations/ OR Paranoid Disorders/ OR Dissociative Disorders/
11	(delusion* or hallucin* or paranoi* OR psychosis OR psychoses OR psychotic* OR schizo* OR dissociation OR dissociative).ti,ab,kf,ot,oa.
12	exp Dyssomnias/ OR exp Parasomnias/
13	((sleep* adj1 (disorder* OR disturb* OR depriv* OR apnea OR syndrome* OR initiat* OR maintenance OR paralysis)) OR insomni* OR dyssomni* OR sleepless* OR hypersominia OR hypersomnolence OR narcolep* OR cataple* OR somnambulism OR "sleep walk*" OR sleepwalk* OR "sleep wake" OR parasomni*).ti,ab,kf,ot,oa.
14	Sexual Dysfunctions, Psychological/ OR Dyspareunia/ OR "Sexual and Gender Disorders"/ OR Vaginismus/
15	((sex* OR psychosex* OR orgasm* OR arousal) adj2 (disorder* OR dysfunction*) OR dyspareuni* OR vaginism* OR "sex* aversion").ti,ab,kf,ot,oa.
16	exp Self-Injurious Behavior/
17	(suicid* OR selfharm* OR "self harm*" OR selfinjur* OR "self injur*" OR selfmutilat$ OR "self mutilat*" OR selfdestruct* OR "self destruct*" OR selfpoison* OR "self poison*" OR (self adj2 cut*) OR cutt* OR selfimmolat* OR "self immolat*" OR selfinflict* OR "self inflict*" OR automutilat* OR "auto mutilat*" OR nonsuicid* OR "non-suicid*" OR parasuicid* OR "para-suicid*").ti,ab,kf,ot,oa.
18	exp "Feeding and Eating Disorders"/ OR exp Hyperphagia/

19	(anorexi* OR ((appetite OR eating) adj disorder*) OR binge* OR binging OR purg* OR bulimia OR bulimic* OR (compuls* AND (eat* or vomit*)) OR (food* AND bing*) OR hyperphagi* OR ("self induc*" AND vomit*) OR pica OR orthore* OR geophag* OR ednos OR osfed OR arfid OR "avoidant restrictive food intake" OR allotriophag*).ti,ab,kf,ot,oa.
20	exp Substance-Related Disorders/ OR exp Drug Misuse/ OR exp Illicit Drugs/ OR Designer Drugs/ OR "Marijuana Use"/ OR exp Narcotics/
21	(((alcohol* OR drug$1 OR polydrug* OR substance* OR polysubstance* OR liquor*) adj2 (use* OR using OR abus* OR addict* OR misus* OR dependen* OR illegal* OR illicit* OR unlawful* OR criminal* OR overdos* OR recreation* OR "over dos*" OR abstain* OR abstinen* OR consumes OR consume OR consumption OR problem$1 OR nonmedical OR "non-medical" OR criminal*)) OR ((drug* or polydrug* or substance* OR polysubstance*) adj rehab$) OR (crave* adj2 inject*) OR "abusable product*" OR "hard drug" OR "hard drugs" OR "soft drug" OR "soft drugs" OR "needle fixation" OR ATOD OR "binge drinking" OR "drinking binge*" OR "habitual drinking" OR "drinking habit*" OR alcoholism OR (chemical* adj (dependen* OR addict*)) OR ((amphetamin* OR cannabis* OR cocaine OR dexamfetamin* OR dextroamphetamin* OR dexedrine OR heroin OR marijuana OR marihuana OR methamphetamin* OR psychostimulant* OR stimulant$1 OR opioid* OR opiate* OR fentanyl OR benzodiazepine* OR tranquiliz* OR hallucinogen* OR amobarb* OR "angel dust" OR barbiturate* OR demerol OR hash OR hashish OR hydrocodone OR hydromorphone OR lsd OR "lysergic acid" OR mdma OR morphine OR narcotic* OR opium OR oxycodone OR oxycontin OR ecstasy OR pcp OR "street drug*" OR vicodin OR ketamine OR inhalant OR psychedelic*) adj2 (abus* or addict* or misus* or depend* or use$1))).ti,ab,kf,ot,oa.
22	exp Psychotropic Drugs/
23	((psychiatric OR psychological) adj2 (hospital* OR service* OR outpatient* OR inpatient* OR patient* OR treatment* OR admission*)).ti,ab,kf,ot,oa.
24	((antidepress* OR "anti-depress*" OR antianxiety OR "anti-anxiety" OR antipsychotic* OR "anti-psychotic*" OR psychotropic* OR stimulant* OR psychoactive OR antimanic OR "anti-manic") adj3 (drug* OR medicat* OR agent* OR substance*)).ti,ab,kf,ot,oa.
25	or/ 4-24
26	3 AND 25

Websites to be searched:

- AbortionRisks /Thomas W. Strahan Memorial Library: https://abortionrisks.org/index.php/Main_Page
- Abortion Clinical Research Network: https://societyfp.org/research/abortion-clinical-research-network/
- Alliance for Post-Abortion Research and Training (APART): https://standapart.org/
- American Association of Pro-Life ObGyns: https://aaplog.org/
- American College for Obstetrics and Gynecology: https://www.acog.org/
- American Medical Association: https://www.ama-assn.org/
- American Psychiatric Association: https://www.psychiatry.org/
- American Psychological Association: https://www.apa.org/
- ANSIRH: https://www.ansirh.org/research/ongoing/turnaway-study; https://www.ansirh.org/
- Association for Interdisciplinary Research in Values and Social Change: https://www.nrlc.org/proliferesearch/
- British Pregnancy Advisory Service: https://www.bpas.org/
- Brookings Institute: https://www.brookings.edu/
- Center for AmericanProgress: https://www.americanprogress.org/
- Centers for Disease Control: www.cdc.gov
- Center for Reproductive Rights: https://reproductiverights.org/
- Child Trends: https://www.childtrends.org/
- ClinicalTrials.gov: https://www.clinicaltrials.gov/
- Elliott Institute: http://www.elliotinstitute.org/
- Family Research Council: https://www.frc.org/
- Guttmacher Institute: https://www.guttmacher.org/
- Heartbeat International: https://www.heartbeatinternational.org/
- Heritage Foundation: https://www.heritage.org/
- Ibis Reproductive Health: https://www.ibisreproductivehealth.org/research-areas/abortion
- International Union for the Scientific Study of Population: https://iussp.org/en/panel/abortion-research
- Kaiser Family Foundation: https://www.kff.org/
- Life Issues Institute: https://lifeissues.org/
- Lozier Institute: https://lozierinstitute.org/
- National Right to Life: https://www.nrlc.org/
- NIH RePORTER: https://reporter.nih.gov/
- Pew Research Center: https://www.pewresearch.org/
- Planned Parenthood: https://www.plannedparenthood.org/
- Population Council: https://popcouncil.org/
- Project Rachel After Abortion: https://helpafterabortion.org/en/home
- Project Rachel Ministry: https://hopeafterabortion.com/
- Rachel’s Vineyard: https://www.rachelsvineyard.org/
- PRRI: https://www.prri.org/topic/abortion-reproductive-health/
- Silent No More: https://www.silentnomoreawareness.org/
- Society of Catholic Social Scientists: http://www.catholicsocialscientists.org/
- The Unchoice: http://www.theunchoice.com/pblresearch.htm
- UK Royal College of Physicians: https://www.rcplondon.ac.uk/
- US National Academies Press: https://nap.nationalacademies.org/
- Urban Institute: https://www.urban.org/
- World Expert Consortium for Abortion Research and Education (WECARE): https://www.wecareexperts.org/
- World Health Organization: https://www.who.int/

Appendix 2. Screening and eligibility tool

Screening of titles and abstracts (Stage 1) and eligibility decisions (Stage 2) are conducted in Covidence.

Stage 1: Initial screening of titles and abstracts (based on pilot tests involving duplication screening of 150 titles and abstracts)

Screening rules and conventions

- Screening decisions
  - Yes = appears to meet inclusion criteria
  - No = meets exclusion criteria
  - Maybe = unclear
- We do not make screening decisions based on a document title alone, because titles do not provide enough information to make well-informed screening decisions.
- If there is no English-language abstract in Covidence, we will skip screening until the abstract (or a portion of the document) is located, translated into English if necessary, and uploaded to Covidence.
- Outcomes may not be clearly defined in titles and abstracts, so we do not apply *outcome inclusion criteria* when screening titles and abstracts. We can apply some outcome *exclusion* criteria (e.g., focus only on physical effects of abortion) in screening decisions.
- After double-screening titles and abstracts, we promote documents to full-text review if there are: conflicting scores, or 2 Yes, or 2 Maybe votes. Both reviewers have to say No in order to halt further consideration of a title and abstract.
- Reviews of potentially relevant research, as well as commentaries and editorials that may contain relevant references, should be *tagged* in Covidence as "Relevant reviews" and accompanied by No votes.
- We will also *tag* documents that appear to be related to potentially eligible studies if those documents do not otherwise meet our screening criteria (e.g., descriptive or implementation studies, corrections, retractions). These documents should receive No votes if they do not meet screening criteria, but should be tagged with "Related to potentially eligible study".

Screening criteria for titles/abstracts

Phrases and sections in brackets { } will be used when screening titles and abstracts, and will be dropped for eligibility decisions based on full text.

	Inclusion Criteria	Exclusion Criteria
Study Characteristics	• Cross-sectional, longitudinal, or case-control study<br>• Study with two or more parallel cohorts (non-overlapping groups) of people who were assessed at the same point(s) in time {or unclear}<br>• Two or more parallel cohorts were in the same location(s) (i.e., countries, regions, municipalities with shared legal permissions/restrictions on abortion) {or unclear}<br>• Study protocols that meet other inclusion criteria	• Review article (tag)<br>• Qualitative study, case study, or single group study<br>• Theoretical or position paper, editorial, or book review<br>• Practical guidelines or treatment manual<br>• Historical comparisons, geographic comparisons, comparisons of

		different types of abortion<br>● Studies not based on individual participant data
Population	● People who could become pregnant (cisgender women, transgender men, and gender non-binary individuals who are capable of pregnancy and childbirth)	● People who cannot become pregnant
Intervention / Exposure	● Abortions that use approved medications (mifepristone and/or misoprostol, and methotrexate) and/or medical procedures intended to terminate pregnancy.<br>● Self-managed medication abortions are included.<br>● Procedural abortions are included if they were performed by health care professionals.	● Multifetal pregnancy reduction<br>● Use of other substances (herbs, drugs) or non-medical procedures (including physical force) to induce abortion
Comparator / Context	● Any group that can (or did) become pregnant but did not have an abortion.<br>● Includes childbirth (full term or premature delivery), fetal loss (miscarriage or stillbirth), and no pregnancy.	● People who cannot become pregnant<br>● We are not comparing medical to procedural abortions
Outcomes	[Outcome inclusion criteria are reserved for full text eligibility decisions]	● Studies related only to physical effects of abortion (e.g., medical complications, infection, uterus perforation)
Other	● All authors, institutions, geographic regions, settings, languages, reports dates, report types (regardless of publication status or peer review status), and all follow-up data.	● Studies of nonhuman animals

Stage 2: Eligibility decision (based on reading of all relevant full text reports)

1. **Study design**: Does the study include:
   a. two or more parallel cohorts (non-overlapping groups) of people who were

   b. assessed at the same point(s) in time AND

   c. in the same location(s) (i.e., countries, regions, municipalities with shared legal permissions/restrictions on abortion)?

   - Yes
   - No [STOP; the study is excluded for this reason, describe the study design]
   - Unclear

2. **Population:** Does the study only include people who could become pregnant?
   - Yes
   - No [STOP; the study is excluded for this reason, describe the study sample]
   - Unclear

3. **Interventions:** Does the study include people who use approved medications (mifepristone and/or misoprostol, and methotrexate) and/or medical procedures intended to terminate pregnancy? (Self-managed medication abortions are included. Procedural abortions are included only if they were performed by health care professionals.)
   - Yes
   - No [STOP; the study is excluded for this reason, describe the interventions]
   - Unclear

4. **Comparison:** Does the study include one or more groups of people who did not have abortions?
   - Yes
   - No [STOP; the study is excluded for this reason, describe the comparisons]
   - Unclear

5. **Type of outcomes**: Did the study **measure** mental health outcomes?
   - Yes
   - No [STOP; the study is excluded for this reason, describe the outcomes measured]
   - Unclear

6. **Reverse time order:** Were mental health data **only** collected **before** the abortion (or end of pregnancy or similar point in time on the calendar for comparison cases)?
   - Yes [STOP; the study is excluded for this reason]
   - No
   - Unclear

Appendix 3. Data extraction tool

Study ID ___ (eligible studies only)
Coder Initials ____
Date DDMMYYY

3. Dataset information

3.1. Does this study come from a larger (potentially shared) dataset?

- Yes (identify the name of the dataset)
- No
- Unclear (explain)

4. Study-level data

Research methods

4.1. Original data sources (check all that apply)

- Structured surveys (in-person interviews, telephone interviews, self-administered survey, computer-assisted survey)
- Repeated measures (number of data collection points)
- Retrospective measures
- Registry or administrative data (identify and describe)
- Other (explain)

4.2. Number of parallel groups:

- Number of distinct abortion groups (if > 1, explain differences)
- Number of different comparison groups (if > 1, explain differences)

4.3. Type(s) of comparison groups (check all that apply):

- Sought but did not receive abortions
- Unintended or unwanted pregnancies (explain how unintended or unwanted pregnancy was defined and measured)
- All pregnancies that did not end in abortion
- Persons capable of pregnancies
- Other (explain)

4.4. Methods used to control for initial differences between groups on age, socioeconomic status, and initial mental health variables:

- Case controls
- Propensity score matching
- Other matching procedures (explain)
- Statistical controls (e.g., OLS regression, logit or logistic regression, partial correlation, hierarchical models)
- Other (explain)
- None

4.5. Timing of data collection (check all that apply)

- before pregnancy
- during pregnancy
- < 2 weeks after pregnancy ended
- 14 to 90 days after pregnancy ended
- 90+ days after pregnancy ended

Funding and potential conflicts of interest

4.6 Funding sources for the study (describe)

4.7. Did the investigators declare any potential conflicts of interest (COIs)? Y/N
If Yes, what kinds of conflicts were declared?

- Personal (explain)
- Professional (explain)
- Financial (explain)
- Other (explain)

4.8. Are there any foreseeable financial, reputational, or personal benefits to investigators if results favored the abortion group or the comparison group?

- Yes (describe)
- Unclear

Time and location

4.9. Enrollment timing
4.9.1. Date on which enrollment into the study began: DDMMYYY
4.9.2. Date on which enrollment into the study ended: DDMMYYY
4.9.3. Date of most recent follow-up: DDMMYYY

4.10. Location: Country, State/Province, City or metro area

4.11. Does the study provide any information on legal restrictions on abortion at the time and location of the study? Yes/No

4.11.1. If Yes, what kinds of restrictions, if any, were in place? (select all that apply)

- No restrictions
- Parental consent required for minors
- Counseling required (describe)
- Waiting period required (describe)
- Restrictions linked to gestational duration (describe)
- Abortion banned with some exceptions (describe exceptions)
- Abortion banned, no exceptions
- Other (explain)
- Unclear

4.12. Stigma: is there any available information on perceived stigma related to abortion at the time and location of the study?

- Yes (explain)
- No
- Unclear

Participant characteristics

4.13. Sample eligibility requirements (describe) _______

4.14. Sampling recruitment methods (explain) _______

4.15. Aside from abortion, were the same inclusion criteria applied to both groups? Y/N/Unclear

4.16. Were the same exclusion criteria applied to both groups (e.g., subsequent abortions)?

- Yes
- No (explain)
- Unclear (explain)

Cohort sizes

Number of cases	Abortion cohort(s)	Other cohort(s)	Total	Pg# & Notes
Consented to participation				
Provided initial data on mental health measures (prior to end-of-pregnancy event)				
Completed at least one outcome measure				
Completed latest follow-up (outcome measure)				

Demographic and personal characteristics

		Abortion cohort(s)	Other cohort(s)	Total sample	Pg# & Notes
Participant age at study enrollment	Mean, sd, min, max				
Race/ethnicity	% White				
	% Black				
	% Hispanic/Latinx				
	% Asian/Pacific				
	% Other				
Socioeconomic status	% completed high school				
	% completed college				
	% unemployed				
	% in poverty				
	% receive public aid				
	median income or other measure				
Family composition, previous pregnancies and births	% single parent				
	# previous pregnancies (mean, sd, min, max)				
	# previous live births (mean, sd, min, max)				
	# of children in household (mean, sd, min, max)				
Abortion history	# previous abortions (mean, sd, min, max)				
Early life trauma (e.g., ACES)	instrument used, mean, sd, min, max				
Exposure to violence	instrument used, mean, sd, min, max				
Social support (general)	instrument used, mean, sd, min, max				
Social support (for the abortion decision)	instrument used, mean, sd, min, max				
Perceived stigma of abortion	instrument used, mean, sd, min, max				

4.17. Were there any significant differences between cohorts on demographic characteristics (p-value < .05 and/or d > 0.25)? [IF NECESSARY, CALCULATE d USING ES_Calculator.xls]

- Yes (describe differences, using p and/or d values)
- No (how do we know?)
- Unclear (p and d not provided or cannot be calculated)

Prior mental health characteristics

4.18. Type of data available (select one):

- No data available [SKIP TO Pregnancy characteristics]
- Retrospective reports only (describe recall period, i.e., lag time)
- Mental health data collected prior to abortion and at similar time for comparison cases

		Abortion corhort(s)	Other cohort(s)	Total sample	Pg# & Notes
Overall mental health symptoms	Mean, sd, min, max				
Specific mental health condition (describe)	Mean, sd, min, max				
	% meet diagnostic criteria				
	% self-report				
Specific mental health condition (describe)	Mean, sd, min, max				
	% meet diagnostic criteria				
	% self-report				
Specific mental health condition (describe)	Mean, sd, min, max				
	% meet diagnostic criteria				
	% self-report				

4.19. Were there any significant differences between cohorts on mental health characteristics at baseline (p-value < .05 and/or d > 0.25)? [IF NECESSARY, CALCULATE d USING ES_Calculator.xls]

- Yes (describe differences using p and/or d values)
- No (how do we know?)
- Unclear (p and d not provided or cannot be calculated)

4.20. Were there any significant differences on baseline mental health measures between participants who completed follow-up assessments and those lost to follow-up (p< .05 and/or d > 0.25)? [IF NECESSARY, CALCULATE d USING ES_Calculator.xls]

- Yes (describe differences using p and/or d values)
- No (how do we know?)
- Unclear (p and d not provided or cannot be calculated)

4.21. Pregnancy characteristics

	Abortion cohort(s)	Other cohort(s)	Total	Pg# & Notes
% with unwanted or unintended pregnancies [1]				
% with fetal anomalies [2]				
% with medical complications [2]				
gestational duration in weeks at end of pregnancy (mean, sd, min, max)				

[1] Describe investigators' definition/measure of pregnancy wantedness/intention (unplanned, unwanted, and/or mistimed).
[2] Describe the nature of fetal anomalies or medical complications related to pregnancy, if applicable. Were any comparison cases forced to carry these pregnancy to term? (if so, how many?)

4.22. Abortion characteristics (if available)
- n/% with medication abortions (describe sources and types of medication, if provided)
- n/% with self-managed medication abortions (which aspects of abortion were self-managed?)
- n/% with procedural abortions (describe procedures used, if provided)
- n/% other (explain)
- n/% unclear

4.23. Was abortion self-reported?
- Yes
- No (explain how abortion was reported)
- Unclear

4.24. Other cohort(s) reproductive events
- n/% with live births
- n/% with stillbirths
- n/% with miscarriages
- n/% self-induced or other non medication or nonmedical abortion
- n/% sought but were denied abortion
- n/% not pregnant
- n/% other (explain)

4.25. Were other reproductive outcomes self-reported?
- Yes
- No (explain these outcomes were reported)
- Unclear

5. Risk of bias assessment (study level)

Instructions: complete this section *after* completing data extraction on measurement instruments and effect sizes (below). Responses to questions 5.1 to 5.6 could be entered and/or displayed in a confounder matrix, shown in Figure 2.

5.1. Time order: study design establishes a clear temporal order of abortion and mental health measures.
- Yes = Low risk: longitudinal study collected data on mental health measures before and/or after abortion or other pregnancy events (or at a similar point in time on the calendar for comparison cases).
- Unclear risk = insufficient information.

- No = High risk: use of correlational data and/or lifetime measures of abortion and/or mental health outcomes.

5.2. Avoidance of confounding (initial equivalence on mental health history): initial differences between groups on measures of mental health history were small or moderate (d ≤ 0.25) or researchers used statistical controls (e.g., propensity score matching, regression covariates) for initial differences.

- Yes = Low risk
- Unclear risk: insufficient information (e.g., group-level data were not provided, d cannot be computed, unclear if statistical controls were sufficient to create comparable groups)
- No = High risk: there were initial differences between groups with d > 0.25, no/inadequate statistical controls for these differences, or no measures of mental health history.

5.3. Avoidance of confounding (pregnancy intention): comparison groups were matched on pregnancy intention (e.g., pregnancy intention was used as an inclusion criterion, used in matching designs and/or the proportion of cases with unintended or unwanted pregnancies was equivalent (d ≤ 0.25) across groups).

- Yes = Low risk.
- Unclear risk = insufficient information.
- No = High risk: between group differences on pregnancy intentions (d > 0.25).

5.4. Avoidance of confounding (initial equivalence on background characteristics): initial differences between groups on socioeconomic variables (e.g., income, education, employment) and family background characteristics (e.g., adverse childhood experiences, interpersonal violence) were small or moderate (d ≤ 0.25) or researchers used statistical controls (e.g., propensity score matching, regression covariates) for baseline differences.

- Yes = Low risk
- Unclear risk: insufficient information (e.g., group-level data were not provided, d cannot be computed, unclear if statistical controls were sufficient to create comparable groups)
- No = High risk: there were initial differences between groups with d > 0.25, and no/inadequate statistical controls for these differences.

5.5. Avoidance of confounding (other criteria): comparison groups were constructed and maintained with similar inclusion and exclusion criteria (e.g., related to age, prior pregnancies, live births, abortions, and prior mental health status), taking into account any country- or region-specific regulations limiting access to abortion to certain groups.

- Yes = Low risk.
- Unclear risk = insufficient information.
- No = High risk: inclusion or exclusion criteria were applied differently to different comparison groups.

5.6. Avoidance of confounding (performance bias): there were no systematic differences (d ≤ 0.25) between groups (or use of statistical controls for differences between groups) in terms of levels of service, care, attention, perceived stigma, or social support.

- Yes = Low risk
- Unclear (insufficient information)
- No = High risk: one group received more services, care, attention, perceived stigma, or support and these factors were not accounted for in the analysis.

5.7. Avoidance of detection bias (blinding): assessor is unaware of group membership when collecting outcome data.

- Yes for all outcomes = Low risk
- Yes for some outcomes = Unclear
- Unclear (insufficient information)
- No = High risk

5.8. Avoidance of attrition bias: Losses to follow up were less than or equal to 25% and equally distributed ($\leq$ 10% difference in response rates) across groups. Group equivalence on important baseline characteristics was retained after losses to follow-up (d $\leq$ 0.25).

- Yes for all outcomes = Low risk
- Yes for some outcomes = Unclear
- Unclear (insufficient information)
- No = High risk: loss of baseline equivalence (d > 0.25), losses to follow up > 25% overall, or losses were unequally distributed (>10% difference) across groups.

5.9. Standardized observation periods: follow-up data were collected from each case at a fixed point in time (e.g., after the end of pregnancy), or analyses included statistical controls for variable observation periods (e.g., event history analysis).

- Yes for all outcomes = Low risk
- Yes for some outcomes = Unclear
- Unclear (insufficient information)
- No = High risk

5.10. Validated measures of mental health history: use of instruments with demonstrated reliability (e.g., alpha/kappa > .7) or validity in the study sample and/or similar samples from (similar countries, socioeconomic, and racial/ethnic groups), or use of external administrative data on events (e.g., psychiatric hospitalization).

- Yes for all outcomes = Low risk
- Yes for some outcomes = Unclear
- Unclear (insufficient information)
- No = High risk

5.11. Validated measures of mental health outcomes: use of instruments with demonstrated reliability (e.g., alpha/kappa > .7) or validity in the study sample and/or similar samples from (similar countries, socioeconomic, and racial/ethnic groups), or use of external administrative data on events (e.g., psychiatric hospitalization).

- Yes for all outcomes = Low risk
- Yes for some outcomes = Unclear
- Unclear (insufficient information)
- No = High risk

5.12. Free of selective reporting: a prospective study protocol is available and all pre-specified outcomes are reported in the pre-specified way; all expected outcomes are reported in full and for all cases (e.g., no systematic exclusion of subgroups of cases), regardless of the direction and statistical significance of results.

- Yes = Low risk
- Unclear (e.g., prospective protocol is not available, or changes in the protocol were made after the study began)
- No = High risk: some outcomes are not reported or are reported incompletely (e.g., non-significant results are mentioned, but data are not provided; data are provided for selected subgroups only).

6. Measurement level data: Mental health outcomes

Complete for each measure (of any of our primary and secondary outcomes) used in the study, regardless of whether results were reported for that measure.

6.1 Name of measure
6.2 Measure ID number (assign)

6.3. Outcome domain (check one)
- Post-traumatic stress symptoms or disorder (PTSD)
- Other anxiety disorders or symptoms
- Depression and other mood disorders or symptoms
- Suicide, suicidality, and suicide attempts
- Substance use and substance use disorder
- Any mental disorder or overall mental health symptoms
- Eating disorders
- Sleeping disorders
- Sexual disorders
- Psychotic disorders

6.4 Outcome category (check one)
- Diagnosis or status
- Symptoms
- Service use
- Medication use

6.5 Data source
- Self-report (e.g., survey, interview)
- Administrative data (e.g., hospital records)
- Registry data
- Other (explain)

6.6. Reliability/validity data available for the study sample?
- Yes
- No
- Unclear

6.7. Sample reliability/validity results: Kappa or Chronbach's alpha > 0.7?
- Yes
- No
- Unclear

6.8. Reliability/validity data available for similar samples (similar countries, socioeconomic, racial/ethnic groups)?
- Yes
- No
- Unclear

6.9. Other reliability/validity results: Kappa or Chronbach's alpha > 0.7?
- Yes
- No
- Unclear

7. Effect-size level data

7.1. Name of mental health outcome measure (from 6.1)

7.2. Measure ID number (from 6.2)

7.3. How is the endpoint defined?
  7.3.1. for the abortion group? (e.g., days/weeks/months after abortion or unclear)
  7.3.2. for the comparison group? (e.g., days/weeks/months since end of pregnancy, other marker, point in time, or unclear)

7.4. What is the time referent for relevant measures?
  7.4.1. Abortion measure: lifetime, within past year, past 3 months, other
  7.4.2. Mental health measure: lifetime, past year, past 3 months, current

7.5. Type of effect size (ES)
- Dichotomous
- Continuous

7.6. Polarity: High score represents
- More severe symptoms or the presence of a disorder
- Less severe symptoms or no disorder

7.7. Type of ES (detail)
- Unadjusted (zero-order) correlation
- Partial correlation or regression coefficient
- Standardized mean difference (SMD, d, g)
- Odds ratio, risk ratio, or risk difference
- Other (explain)

7.8. Were results adjusted for initial measures and/or covariates?
- Yes
- No
- Unclear

7.9. Control variables used in calculation of this ES (list all).

7.10. Statistical model used in calculation of this ES (describe).

7.11. Enter raw data to support calculation of ES (SMD, OR, RR, RD) or enter correlation, partial correlation (regression coefficients) and standard error.

7.12. Valid Ns for each group (program calculates % attrition and % differential attrition for each ES from initial sample size)

7.13. Were values for missing data imputed for this ES?
- Yes (how? Describe imputation methods used)
- No
- Unclear
